# Postnatal Development of Pyramidal Neurons Excitability and Synaptic Inputs in Mouse Gustatory Cortical Circuits

**DOI:** 10.1523/ENEURO.0329-25.2026

**Published:** 2026-04-28

**Authors:** Hillary Schiff, Arianna Maffei

**Affiliations:** ^1^Department of Neurobiology and Behavior, Stony Brook University, Stony Brook, New York 11794; ^2^Division of Biosciences, College of Dentistry, Ohio State University, Columbus, Ohio 43210; ^3^Graduate Program in Neuroscience, Stony Brook University, Stony Brook, New York 11794

**Keywords:** development, excitability, GABA_A_, insular cortex, parvalbumin, taste

## Abstract

Cortical neurons in sensory areas undergo a protracted process of postnatal maturation that includes changes in membrane properties, synaptic drive, and connectivity. The completion of this process is associated with the closure of critical periods for experience-dependent plasticity in visual, auditory, and somatosensory cortices. Whether these findings extend to the postnatal development of cortical circuits for taste is currently unknown. Taste receptor cells in the taste buds reliably fire action potentials in response to taste stimuli by the third postnatal week and show extended refinement of membrane excitability into adulthood. Taste responsive neurons in the nucleus of the solitary tract show reorganization of peripheral nerve terminals (NTS) over a timeline comparable to taste buds. However, no study to date investigated the postnatal development of neurons in the gustatory cortex (GC). Here, we focused on pyramidal neurons in the deep layers of GC in acute slices from male and female mice and compared their membrane properties from the third to the eighth postnatal week. We report changes in intrinsic excitability and a shift of the excitation/inhibition (E/I) balance toward inhibition as pyramidal neurons reach their young adult properties. The increase in inhibitory drive accompanied a protracted process of postnatal maturation of inhibitory circuits mediated by parvalbumin-expressing neurons (PV^+^ neurons) that showed an increase in their association with perineuronal nets as well as refinement of their connectivity onto pyramidal neurons. Together, our results indicate that GC neurons undergo protracted postnatal maturation that may influence taste response properties.

## Significance Statement

We show that the circuit in the gustatory cortex (GC) undergoes a protracted maturation process extending into adulthood that shifts the excitability of GC toward inhibition through changes in pyramidal neurons membrane properties, increased inhibitory synaptic drive, and refinement of parvalbumin neurons connectivity. GC circuit refinement extends beyond the developmental windows previously reported for other sensory cortical circuits and overlaps with the window of maturation for taste receptor cells and with the critical period for the development of taste preferences. As finding nutritious food sources may require the integration of vision, audition, somatosensation, and olfaction, an extended maturation of GC may facilitate the integration of sensory information for the identification of food and the decision to ingest it.

## Introduction

During postnatal development, mammals shift from relying on their mother's milk to foraging for food. Early experience with feeding independence influences the development of taste preferences (Schiff et al., 2023). While the postnatal development of gustatory cortical circuits is not well studied, there is some experimental evidence for protracted maturation of neuronal morphology and early-life experience-dependent effects on neurons in other regions of the taste system. In mice, taste receptor cells begin to reliably fire action potentials during the third postnatal week (Bigiani et al., 2002) and the refinement of their excitability extends into adulthood (Bigiani et al., 2002; Ohtubo et al., 2012). Postnatal anatomical rewiring was observed in the first central relay in the gustatory system, the nucleus of the solitary tract (NTS) after postnatal day 21 (P21), with the inputs to the NTS reaching adult connectivity by P35 and undergoing additional refinement into adulthood (Hill et al., 1983; Sollars et al., 2006; May et al., 2008; Sun et al., 2017). In the gustatory portion of the parabrachial nucleus, dendritic arborization of multipolar and fusiform cells reach adult morphology by P35 (Lasiter and Kachele, 1988). Together, these studies identify the postnatal window between P15–P21, P21–P35, and P50–P65 as periods of maturation for different circuits in the gustatory system.

In primary visual, auditory, and somatosensory cortices, developmental time windows of heightened sensitivity to changes in sensory inputs extending between the third and fifth postnatal week have been identified (Micheva and Beaulieu, 1995; Antonini et al., 1999; Maffei et al., 2006, 2010; Maffei and Turrigiano, 2008b; Wang et al., 2011; Takesian et al., 2012, 2018; Gainey and Feldman, 2017; Gainey et al., 2018). During these periods, known as critical periods, cortical circuits undergo a maturation process that is shaped by experience and reach their adult properties.

GABAergic inhibitory synapses in particular play a crucial role in postnatal cortical circuit maturation and refinement. Inhibitory cortical circuits themselves undergo extended postnatal maturation (Hensch, 2004; Tatti et al., 2017; Takesian et al., 2018), with increases in GABAergic inhibition opening the critical period for circuit refinement. For instance, in a knock-out mouse in which GABA is severely diminished (GAD-KO), the critical period may never open unless GABA receptors are activated pharmacologically (Fagiolini and Hensch, 2000). Changes in inhibitory circuits during critical periods are primarily ascribed to parvalbumin-expressing interneurons (PV^+^ INs). Reports show an increase in the number of PV^+^ INs (Gonchar et al., 2007; Tatti et al., 2017) along with increased perisomatic innervation of pyramidal neurons (Chattopadhyaya et al., 2004). This process is associated with increases in the expression of PV in PV^+^ INs (Murase et al., 2017), and the duration of the critical period correlates with the accumulation of perineuronal nets (PNNs) around PV^+^ INs somata (Sorg et al., 2016).

In adult gustatory cortex (GC), inhibitory responses make up a component of the complex taste-evoked responses (Yamamoto, 1984) and shape the excitability of pyramidal neurons. Furthermore, feedforward inhibition mediated by PV^+^ INs is recruited by both thalamocortical (Haley et al., 2023) and basolateral amygdala inputs (Haley et al., 2016), suggesting that this population of inhibitory neurons gates incoming inputs and shapes the response properties of GC pyramidal neurons. Recent work identified a critical period for taste experience-dependent plasticity that depends on the maturation of PV^+^ INs in GC (Schiff et al., 2023) and extends from the third postnatal week to the end of the eighth postnatal week. This suggests that despite significant differences in circuit organization (Haley et al., 2016, 2023), GC may share some mechanisms of postnatal maturation with other sensory cortices.

In this study, we used morphological reconstructions, whole-cell patch-clamp electrophysiology in acute slice preparations, immunohistochemistry, and channelrhodopsin-assisted circuit mapping (CRACM; Petreanu et al., 2007) to track postnatal changes in the membrane properties of deep layer pyramidal neurons and assess possible age-dependent refinement of the connectivity of GC PV^+^ INs of male and female mice during the postnatal window straddling the transition to independent feeding and adulthood. Pyramidal neurons showed a transient decrease in excitability and a shift in the excitation/inhibition (E/I) balance toward inhibition mediated by an increase in inhibitory synaptic drive with no overall change in excitation. The changes in synaptic inhibition were accompanied by increased PV fluorescence intensity, accumulation of PNNs around PV^+^ INs, and refinement of the connectivity of PV^+^ INs around the perisomatic region of pyramidal neurons. Our results point to a dynamic refinement process occurring from P17 to P56, a period that overlaps with the maturation of taste receptor cells (Bigiani et al., 2002; Ohtubo et al., 2012), refinement of brainstem circuits for taste (Hill et al., 1983; Sollars et al., 2006; May et al., 2008; Sun et al., 2017), and the critical period for the development of sweet taste preference (Schiff et al., 2023).

## Materials and Methods

### Animals

All experimental procedures followed the guidelines of the National Institutes of Health and were approved by the Institutional Animal Care and Use Committee. Mice of both sexes were group-housed in a vivarium on a 12 h light/dark cycle. Experiments were performed during the light cycle. Wild-type C57BL/6 mice were purchased from Charles River Laboratories, arriving to our facility either as adults or as a litter consisting of the lactating mother and pups at P5. PV-cre (JAX stock #017320; Taniguchi et al., 2011) female and Ai14 (RRID: IMSR_JAX:007908) male mice, both homozygous, were ordered from Jackson Laboratory and crossed in our animal facility. The Ai14 line expresses tdTomato in a cre-dependent fashion (Madisen et al., 2010). The PV-cre;Ai14 offspring were heterozygous for both cre and lox-stop-lox-tdTomato alleles and were used in CRACM experiments.

### Stereotaxic surgery for CRACM

Mice aged P14 ± 1 or P49 ± 1 were deeply anesthetized with a cocktail of 100 mg/kg ketamine and 10 mg/kg xylazine injected intraperitoneally. Once anesthetized, mice were placed on the stereotaxic apparatus and received an injection of bupivacaine (2.5 mg/ml, ∼0.1 ml) under the scalp for local anesthesia. A craniotomy was made over the left GC at coordinates adjusted for developmental stage (P14: +0.9 mm anterior to bregma, +3.05 mm lateral from bregma; P49: +1.0 mm anterior to bregma, +3.25 mm lateral from bregma). A pulled glass micropipette filled with mineral oil was attached to a Nanoject II (Drummond) and backfilled with a solution containing a viral construct (AAV9-hSyn-FLEX-soCoChR). To reach the appropriate titer of viral particles, the stock virus was diluted 1:5 in sterile saline. The tip of the glass micropipette was slowly lowered to a depth of 2.3 mm below the pial surface for both age groups. Approximately 250 nl of the virus solution was injected with pressure pulses delivering ∼13.8 nl per pulse over ∼10 min. To ensure localized delivery of the viral construct, the pipette tip was left in place for ∼10 min after the end of the injection and then slowly retracted. The skin over the craniotomy was then sutured closed and covered with Vetbond. Mice recovered on a heating pad until ambulatory at which point they were returned to the home cage.

### Electrophysiology

To prepare acute coronal slices (350 µm) containing GC, mice were anesthetized with isoflurane using the bell jar method and rapidly decapitated. The brain was dissected in ice-cold, oxygenated standard artificial cerebrospinal fluid (ACSF) containing the following (in mM): 126 NaCl, 3 KCl, 25 NaHCO_3_, 1 NaHPO_4_, 2 MgSO_4_, 2 CaCl_2_, 14 dextrose with an osmolarity of 313–317 mOsm with a pH 7.4 when bubbled with carbogen (95% oxygen, 5% carbon dioxide). The brains were secured to a fresh tissue vibratome (Leica VT1000) and were submerged in oxygenated ACSF during the slicing procedure. Slices were recovered in 34°C oxygenated ACSF for 20 min and then brought to room temperature for 30 min before beginning recordings. Individual slices were transferred to the recording chamber mounted on an upright microscope (Olympus BX51WI). Slices were continuously perfused with oxygenated ACSF and maintained at 34°C with an inline heater (Harvard Bioscience) throughout the recording. Whole-cell patch-clamp recordings were obtained from visually identified layer 5 pyramidal neurons under DIC optics using borosilicate glass pipettes with resistance of 3–5 MΩ. The recording equipment included a HEKA amplifier (EPC10 Quadro USB; Heka Elektronik) with integrated board for data acquisition. Signals were sampled at 10 kHz. Cell capacitance was not compensated during the recording. Patchmaster software (Heka Elektronik) was used for data acquisition.

The recording pipette solution contained the following (in mM): 100 K-gluconate, 20 KCl, 10 K-HEPES, 4 Mg-ATP, 0.3 Na-GTP, 10 Na-phosphocreatine, and 0.4% biocytin, pH was adjusted to 7.35 with KOH; osmolarity was adjusted to 295 mOsm with sucrose. Neurons with a series resistance >15 MΩ or those with series resistance changing >20% during recording were excluded from the analysis. Neuron identity and laminar location was confirmed post hoc with fluorescence immunohistochemistry aimed at identifying neuron morphology, verifying laminar location, and assessing lack of expression of the GABA neuron marker GAD67. Intrinsic excitability measurements were obtained from recordings in current clamp while holding neurons at −70 mV in the presence of the following synaptic receptor antagonists: 6,7 dinitroquinoxaline-2,3-dione (DNQX; 20 µm) to block AMPA/kainate receptors; 2-amino-5-phosphonovaleric acid (APV; 50 µm) to block NMDA receptors; picrotoxin (PTX: 20 µm) to block GABA_A_ receptors. Current steps (700 ms) of increasing intensity (−100 to 400 pA at 50 pA increments) were injected into the cell with a 10 s intersweep interval. The dynamic input resistance (DIR) was calculated as the slope of the current–voltage curve for current injections below action potential threshold. Input–output curves represent the frequency of action potential firing for each suprathreshold current step averaged across recorded neurons for each age group. Rheobase and action potential threshold were quantified from a trace in which the neuron fired a single action potential for two consecutive sweeps. This was accomplished by injecting current steps (700 ms) with increasing amplitude (2 pA steps) around the suprathreshold range. The action potential threshold was measured as the voltage at which the first derivative of the voltage trace reaches 20 mV/ms. For a small number of neurons, it was not possible to isolate two consecutive traces with a single action potential, preventing reliable rheobase and AP threshold measurements. This is reflected in the reported number of neurons used for statistical comparisons in the Results section.

To record spontaneous synaptic currents, slices were perfused with an oxygenated ACSF solution optimized to facilitate spontaneous activity (Maffei et al., 2004; in mM): 124 NaCl, 3.5 KCl, 26 NaHCO_3_, 1.25 NaHPO_4_, 0.5 MgCl_2_, 1 CaCl_2_, 14 dextrose, maintained at 34°C with an inline heater (Harvard Bioscience). Recordings were obtained in voltage clamp with an internal solution containing the following (in mM): 20 KCl, 100 Cs_2_SO_4_, 10 K-HEPES, 4 Mg-ATP, 0.3 Na-GTP, 10 Na-phosphocreatine, 0.2% biocytin (Vrev Cl^−^ = −49.3 mV). The pH was adjusted to 7.35 with KOH and the osmolarity was adjusted to 295 mOsm with sucrose. The sodium channel blocker QX314 (3 mM, Tocris Bioscience) was added to the internal solution to stabilize recordings during prolonged depolarizations. Spontaneous postsynaptic excitatory and inhibitory currents (sEPSCs and sIPSCs, respectively) were recorded by holding neurons at three different holding potentials around the expected reversal for chloride (in mV: −55, −50, −45) and for AMPA/NMDA receptor-mediated currents (in mV: +5, +10, +15). Post hoc current versus holding voltage (Vhold) functions were used to identify the holding voltage that best isolated sEPSCs and sIPSCs, respectively. Traces recorded at the optimal holding voltage were then used for analysis of amplitude and frequency of spontaneous events (Maffei et al., 2004; Maffei and Turrigiano, 2008a). The analysis of spontaneous current was done using Clampfit software (Molecular Devices). Individual postsynaptic events were identified using the template search method (Clements and Bekkers, 1997). Cumulative distributions for sEPSC amplitude and frequency included 35 events/cell, while cumulative distributions for sIPSCs included 100 events/cell. The difference in number of events included in the cumulative distributions was due to the overall lower frequency of sEPSCs compared with sIPSCs in layer 5 GC pyramidal neurons. These events were averaged and rise, and decay time constants for the average sEPSC and sIPSC were calculated for each cell. Total charge was calculated with custom scripts in Igor Pro (WaveMetrics) as the area under the curve for a recording period of 5 min.

### Channelrhodopsin-assisted circuit mapping

A virus coding for a cre-dependent soma-restricted channelrhodopsin (AAV9-hSyn-FLEX-soCoChR-GFP, titer ∼1 × 10^13^ vg/ml, Addgene, catalog #107712-AAV9) was injected into the left GC of PV-cre;Ai14 mice and allowed to incubate for 1 week after verification that this was the optimal time to allow for sufficient expression of the opsin while maintaining soma restriction. The viral construct pAAV-hSyn-FLEX-soCoChR-GFP was a gift from Edward Boyden (Addgene, viral prep #107712-AAV9). Red fluorescence in PV^+^ neurons allowed us to direct our recordings to an area where PV^+^ neurons were present. Initial tests informed us that expression was very low in P17 mice following virus injection at P10, consistent with the small number of PV^+^ INs quantified at this age with immunohistochemistry (Fig. 4). We reasoned that this occurred because PV expression and the number of PV^+^ neurons were too low for sufficient cre expression and thus cre-dependent recombination and expression of the viral products. After assessing that intrinsic properties at P21 were comparable to those measured at P17 (Extended Data Fig. 5-1), we tested the connectivity from PV^+^ INs onto pyramidal neurons at P21 as an early age group in which there was sufficient PV^+^ expression. Virus injection for this group of mice was at P14 ± 1 and recordings at P21 ± 1. For the analysis of PV^+^ IN connectivity onto layer 5 pyramidal neurons, we focused on the earliest and latest age groups. For the older age group, stereotaxic surgery for virus injection was at P49 ± 1 and recordings at P56 ± 1.

We recorded from visually identified layer 5 neurons that did not display red fluorescence and were putatively PV^−^. Post hoc fluorescent immunohistochemistry for biocytin confirmed that these neurons were negative for tdTomato, and their location and morphology was used to confirm that they were indeed layer 5 pyramidal neurons. We delivered patterned light stimulation using a digital micromirror device (Polygon DMD device, Mightex) to focus light from a blue LED (473 nm) onto ∼100 × 100 µm square regions of interest (ROIs) through a 4× objective (Olympus). Pulses were delivered at 1 Hz in an ordered sequence of 5 × 7 ROIs spanning the cortical column surrounding the recorded neuron (∼500 µm dorsal to ventral and 700 µm lateral to medial beginning at the border of layers 1 and 2 and extending to the white matter/corpus callosum). Pulse duration was 5 ms. Light intensity was determined by initial stimulation of the full field of view using an intensity that elicited a reliable postsynaptic response. We collected 10 sequences of 5 × 7 ROI stimulation and averaged the traces in each ROI to determine the magnitude of the IPSC evoked in each illumination region. Although 35 ROIs were stimulated, analyses were conducted on 30 ROIs because the last 5 ROIs (closest to the white matter) often stimulated outside of GC into the claustrum or within the white matter. As none of our recorded neurons responded to stimulation of these 5 ROIs, they were excluded from the analysis. Neurons were considered responsive to stimulation in a specific ROI if an IPSC was elicited in at least 3 out of the 10 sweeps.

### Labeling of recorded neurons

Following electrophysiological recordings, slices were fixed in 4% PFA for 24 h to 1 week. They were then washed in PBS at RT (three times for 5 min) and blocked in 5% NGS and 5% BSA in PBST (1.0% Triton X-100) for 2–4 h at RT. Next, slices were incubated with primary antibodies overnight at 4°C in a solution containing 1% NGS and 1% BSA in PBST (0.1% Triton X-100). The following antibodies were used: streptavidin Alexa Fluor-568 conjugate or streptavidin Alexa Fluor-647 conjugate (1:2000, Invitrogen, S11226 or S32357, respectively), mouse anti-GAD67 (1:500, MilliporeSigma, MAB5406, monoclonal), and/or rabbit anti-RFP (1:500, Abcam 390 004). Sections were then washed with PBS (three times for 10 min) and incubated at RT for 4 h with goat anti-mouse Alexa Fluor 488 (1:200, Thermo Fisher Scientific A-11029) and/or goat anti-rabbit Alexa Fluor 568 (1:200 Thermo Fisher Scientific A-11011), and counterstained with Hoechst 33342 (1:5000, Invitrogen, H3570). After washing with PBS (three times for 10 min), sections were mounted onto glass slides with Fluoromount-G. Sections were imaged with a laser-scanning confocal microscope (Olympus Fluoview) at 10× magnification for validation of location in layer 5 GC and at 40× to determine absence of colocalization with GAD67.

A subset of recorded neurons was first imaged with fluorescent labeling as described above and then unmounted and reprocessed with 3,3′-diaminobenzidine (DAB) for anatomical tracing with Neurolucida. To do this, coverslips were removed from the slides by soaking in PBS and the tissue was removed from the slides. The tissue was rinsed thoroughly in PBS at RT (six times for 10 min). Slices were incubated in 0.3% H_2_O_2_ at 4°C for 1 h and then rinsed in PBS (four times for 10 min). An Avidin-Biotin complex (Vectastain Elite ABC HRP Kit-Vector Labs PK-6100) was kept in the dark for 30 min and then brought up to 10 ml total volume with PBS and 0.1% Triton X-100. Slices were incubated in this solution in the dark at 4°C overnight. The following day, after rinsing four times for 10 min with PBS, slices were preincubated in a DAB solution [Vectastain DAB Peroxidase (HRP) Substrate Kit (with nickel), 3,3′-diaminobenzidine-Vector Labs SK-4100] at room temperature for 15 min, before adding H_2_O_2_ to the well with constant shaking for 40–60 s, until the brown signal was visible by eye. The tissue was quickly rinsed with PBS followed by rinsing four times for 10 min with phosphate buffer (PB). Once mounted on the slides, slices were allowed to dry and then dehydrated through a series of increasing concentrations of ethanol followed by xylene and coverslipped with Entellan medium.

### Immunohistochemistry for PV and PNN labeling

Mice were deeply anesthetized and transcardially perfused with phosphate-buffered saline (PBS), followed by perfusion with 4% paraformaldehyde (PFA) in PBS. Brains were dissected and postfixed in 4% PFA at 4°C for a minimum of 3 h to a maximum of 24 h followed by incubation in PBS-buffered sucrose (30%) solution for cryoprotection until brains were saturated (∼36 h). Thin (50 µm) coronal sections containing GC were cut on a vibratome (VT1000, Leica). Brain sections were first washed in PBS at RT (three times for 5 min) and then incubated in blocking buffer [5% normal goat serum (NGS) and 5% bovine serum albumin (BSA) in PBST with 0.5% Triton X-100] for 1 h at RT. Next, sections were incubated with primary antibodies (mouse anti-PV, 1:2,000, Swant 235, RRID: AB_10000343; biotinylated wisteria floribunda lectin, WFA, 1:500, Vector Laboratories B-1355-2) overnight at 4°C in a solution containing 2% NGS and 1% BSA in PBST (0.1% Triton X-100). Sections were then washed with PBS (three times for 10 min) and incubated with the following fluorescent secondary antibodies at RT for 2 h: goat anti-mouse Alexa Fluor 488 (1:200, Thermo Fisher Scientific A-11029), streptavidin Alexa Fluor 647 (1:500, Thermo Fisher Scientific S32357), and counterstained with neuronal-targeting fluorescent Nissl (NeuroTrace 530/615, 1:200 Thermo Fisher Scientific N21482). After washing with PBS (three times for 10 min), sections were mounted onto glass slides with Fluoromount-G (Southern Biotech). Images were obtained with a laser-scanning confocal microscope (Olympus Fluoview) at 10× magnification.

### PV and WFA imaging analysis

For each animal, we quantified PV and PNN signals in two sections containing GC spaced at 150 µm. The GC outline was previously created and aligned for each section using only the NeuroTrace counterstain to avoid potential bias that might emerge when observing the PV and WFA signal. Quantification of the number of PV^+^ INs and PNNs as well as of fluorescence intensity was obtained in ImageJ using the Pipsqueak AI macro from Rewire Neuro (Sorg et al., 2016). Individual ROIs for each PV^+^ neuron or PNN were automatically created and verified by an experimenter who was blind to the experimental condition. Images from the different fluorescence channels were analyzed separately and then the *x* and *y* coordinates of each ROI were used to determine overlap, which was then confirmed by visual inspection. All single- or double-labeled neurons or PNNs were detected and the mean fluorescence intensity within the ROI was measured. For each image, the average fluorescence intensity across the entire GC region was calculated and was used as background value. The background fluorescence value was subtracted from each ROI fluorescence intensity signal before statistical analysis. This procedure was used to normalize measurements across sections and preparations. The proportion of PV^+^ INs was quantified relative to the fluorescent Nissl counterstain (NeuroTrace). Quantification of NeuroTrace-positive cells was performed using the ImageJ plugin image-based tool for counting nuclei (ITCN; Center for Bio-image Informatics, University of California, Santa Barbara). The group of PV^+^ neurons with a PNN included both neurons’ somata surrounded by mature, high WFA signal intensity PNNs and immature, low WFA signal intensity PNNs. The group of PV^+^ INs lacking PNNs did not show any above-background WFA signal around the somata. The intensity of PV and WFA fluorescence signals were quantified for each ROI in each preparation, normalized to the respective slice background fluorescence and plotted to test for possible correlation between the two parameters in each age group.

### Digital morphological reconstruction

Images of recorded neurons in 350 µm slices reprocessed for DAB-stained were first taken with at 4× magnification on an Olympus BX51WI microscope (Fig. 2*A*). These samples were then digitally reconstructed with Neurolucida (MBF Bioscience) at 40× magnification using a Zeiss Axioplan 2 Imaging (Zeiss ID# M 205234) mounting a motorized stage. Neurons were deemed suitable for reconstruction if there was at least a soma with a tuft of basal dendrites. The soma and all visible basal and apical dendrites were traced. Neurolucida Explorer was used for analysis of each cell for the following parameters: number of basal dendrites, total basal dendrite length, number of basal nodes, total apical dendrite length, number of apical nodes, and convex hull.

### Data analysis

Data are presented as mean ± standard error of the mean (SEM). Electrophysiological analyses for intrinsic properties and opsin-evoked postsynaptic responses were performed using custom scripts written in Igor Pro (WaveMetrics). Cumulative distributions were built after identifying sEPSCs and sIPSCs using the event detection template search in Clampfit (Molecular Devices). Statistical comparisons were made in GraphPad Prism version 10. The statistical tests applied to each dataset are specified in the Results. Statistical significance was determined as a *p* value ≤ 0.05. Correction for multiple comparisons was applied when needed.

## Results

We quantified intrinsic excitability, neuronal morphology, and synaptic drive onto pyramidal neurons in layer 5 of the GC. We focused on this population of neurons as they are primary output neurons of cortical circuits. Recordings were obtained primarily from the granular and dysgranular division of GC, with a few neurons recorded in agranular GC as well. The three divisions of GC can be identified under DIC optics within the same coronal slice by a tapering of layer 4. Hoechst counterstain was used to identify landmarks for borders between cortical regions and divisions. During post hoc image analysis, we applied a mask to each coronal section delineating the boundaries of GC and its subdivisions. This mask was created from sample sections that were immunostained for vGluT2, a marker of thalamic glutamatergic inputs which enabled visualization of layer 4 and Hoechst. Borders were manually traced around GC by comparing our stains and two mouse brain atlases (Paxinos and Franklin, 2001 and the Allen Mouse Brain Atlas). For images of each experimental slice, the mask was aligned laterally to the pial surface, medially to the corpus callosum, and ventrally to the rhinal fissure, allowing consistency across slices in determining into which subdivision the recorded cell belonged. As within each age group the parameters we measured showed no differences across divisions, data were pooled. Pyramidal neurons were visually identified under DIC optics. Upon achieving the whole-cell configuration, the membrane potential of the neuron was set to −70 mV, and square current steps (700 ms) of increasing amplitude (see Materials and Methods) were injected to assess the electrophysiological signature of the recorded neuron and obtain the input/output function in current clamp. To assess synaptic drive, a group of pyramidal neurons was recorded in voltage clamp while holding neurons at the expected reversal potential for excitatory and inhibitory currents. All recorded neurons were filled with biocytin for post hoc morphology assessment using fluorescence microscopy. A subset of recorded neurons was reprocessed for DAB staining and reconstructed with Neurolucida for detailed analysis of neuronal morphology. CRACM (Petreanu et al., 2007) was used to determine postnatal developmental changes in the recurrent connectivity of perisomatic inhibition mediated by PV^+^ neurons.

### Changes in GC pyramidal neurons excitability and morphology during postnatal development

**Figure 1.**
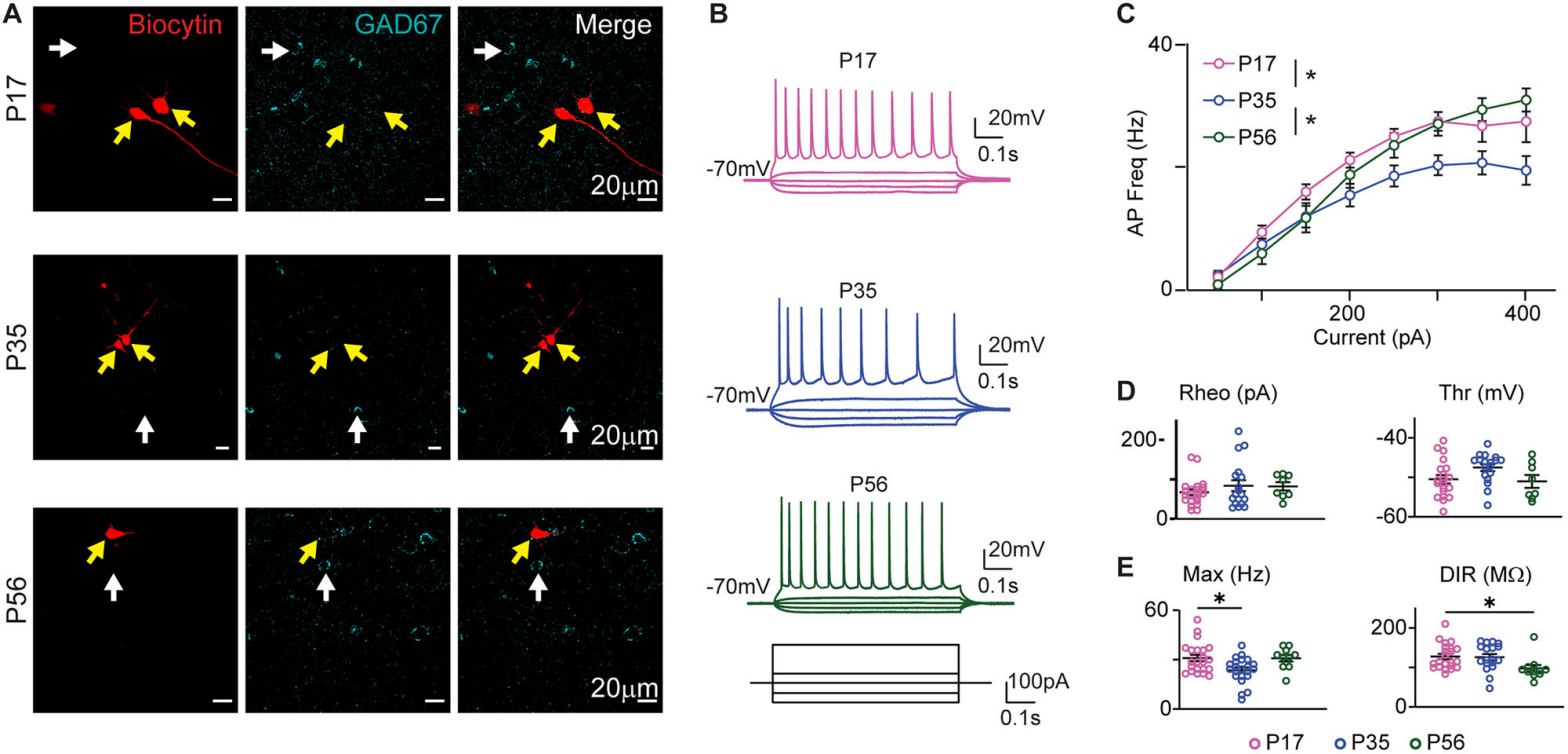
Postnatal developmental changes in intrinsic excitability of GC layer 5 pyramidal neurons. ***A***, Sample single plane confocal images of biocytin-filled recorded neurons showing lack of colocalization with GAD67. The rows show a sample recorded neuron for each age. The yellow arrows point to the recorded neuron filled with biocytin (red); the white arrows point to examples of GAD67^+^ neurons at the same tissue depth. The lack of overlap indicates that the recorded neuron is glutamatergic. ***B***, Sample traces of pyramidal neurons recorded at P17 (pink), P35 (blue), and P56 (green). Bottom, Diagram of step current injections. ***C***, Average input/output function for neurons recorded at each age. Data presented as mean ± SEM. ***D***, Left, Rheobase for each recorded neuron in each age group. Right, Action potential (AP) threshold for each recorded neuron by age group. ***E***, Left, Maximum firing frequency for all recorded neurons plotted by age group. Right, Dynamic input resistance (DIR) quantified as the slope of the input/output function for steps below rheobase. Data are reported as average ± SEM. Pink, P17; blue, P35; dark green, P56. Asterisks, *p* ≤ 0.05.

We began the analysis of pyramidal neurons’ membrane properties by quantifying their intrinsic excitability in the presence of blockers for ionotropic glutamate and GABA receptors. Patch-clamp recordings in the current-clamp configurations were obtained from pyramidal neurons in acute coronal slices containing GC prepared from C57BL/6 mice at P17, P35, and P56 to straddle the postnatal developmental window during which maturation and refinement was reported in other regions of the taste system (e.g., taste buds and NTS; Hill et al., 1983; Bigiani et al., 2002; Sollars et al., 2006; May et al., 2008; Ohtubo et al., 2012; Sun et al., 2017). All neurons included in the analysis were confirmed GC pyramidal neurons in layer 5 as indicated by post hoc fluorescence histological procedures (Fig. 1*A*) and firing patterns (Fig. 1*B*). Input/output functions were built by plotting the frequency of action potentials in response to depolarizing current steps of increasing amplitude (50 pA increments) for neurons recorded in slices from each age group. We observed a transient flattening of the input–output curve between P17 and P35. This effect was reversed in neurons recorded at P56, which showed an input/output function comparable to that of P17 mice [Fig. 1*C*, input–output: P17 *n* = 22 cells from 8 mice; P35 *n* = 22 cells from 11 mice; P56 *n* = 11 cells from 5 mice; *F*_(2,52)_ = 3.85, *p* = 0.028, two-way repeated-measures (RM) ANOVA with main effect of age]. The shift in input/output function was not accompanied by changes in rheobase, nor action potential threshold, consistent with the absence of a lateral shift in the input/output function (Fig. 1*D*, left, rheobase: P17, 67.45 ± 7.23 pA, *n* = 22 cells from 8 mice; P35, 84.11 ± 13.84 pA, *n* = 18 cells from 10 mice; P56, 82.25 ± 10.46 pA, *n* = 8 from 4 mice; *F*_(2,45)_ = 1.761, *p* = 0.4624, one-way ANOVA; right, AP threshold: P17, −50.49 ± 1.06 mV, *n* = 20 cells from 8 mice; P35, −47.53 ± 0.88 mV, *n* = 18 cells from 10 mice; P56, −51.05 ± 1.64 mV, *n* = 8 from 4 mice; *F*_(2,43)_ = 2.843, *p* = 0.0693). However, there was a significant but transient reduction in the maximum action potential firing frequency from P17 to P35, followed by recovery to P17 levels at P56 (Fig. 1*E*, left, max AP firing: P17, 30.91 ± 1.96 Hz, *n* = 22 cells from 8 mice; P35, 23.51 ± 1.69 Hz, *n* = 22 cells from 11 mice; P56, 30.78 ± 1.90 Hz, *n* = 11 cells from 5 mice; *F*_(2,52)_ = 5.33, *p* = 0.01, one-way ANOVA with Tukey's post hoc test: P17 vs P35 *p* = 0.01, P17 vs P56 *p* = 0.99, P35 vs P56 *p* = 0.05). Despite the recovery of the input/output function to P17 levels, the DIR showed a small but significant decrease at P56 when compared with P17 and P35 (Fig. 1*E*, right DIR; P17, 127.60 ± 6.78 MΩ, *n* = 22 cells from 8 mice; P35, 126.20 ± 7.97 MΩ, *n* = 18 cells from 8 mice; P56, 98.71 ± 9.53 MΩ, *n* = 11 cells from 5 mice; *F*_(2,48)_ = 3.721, *p* = 0.032, one-way ANOVA with Tukey's post hoc test: P17 vs P35 *p* = 0.98, P17 vs P56 *p* = 0.04, P35 vs P56 *p* = 0.05). Overall, these results suggest that the intrinsic excitability of GC layer 5 pyramidal neurons decreases during the 5th postnatal week. This effect is transient, as the input/output function and maximum firing frequency return to levels comparable to P17 by early adulthood, despite a significant drop in DIR.

**Figure 2.**
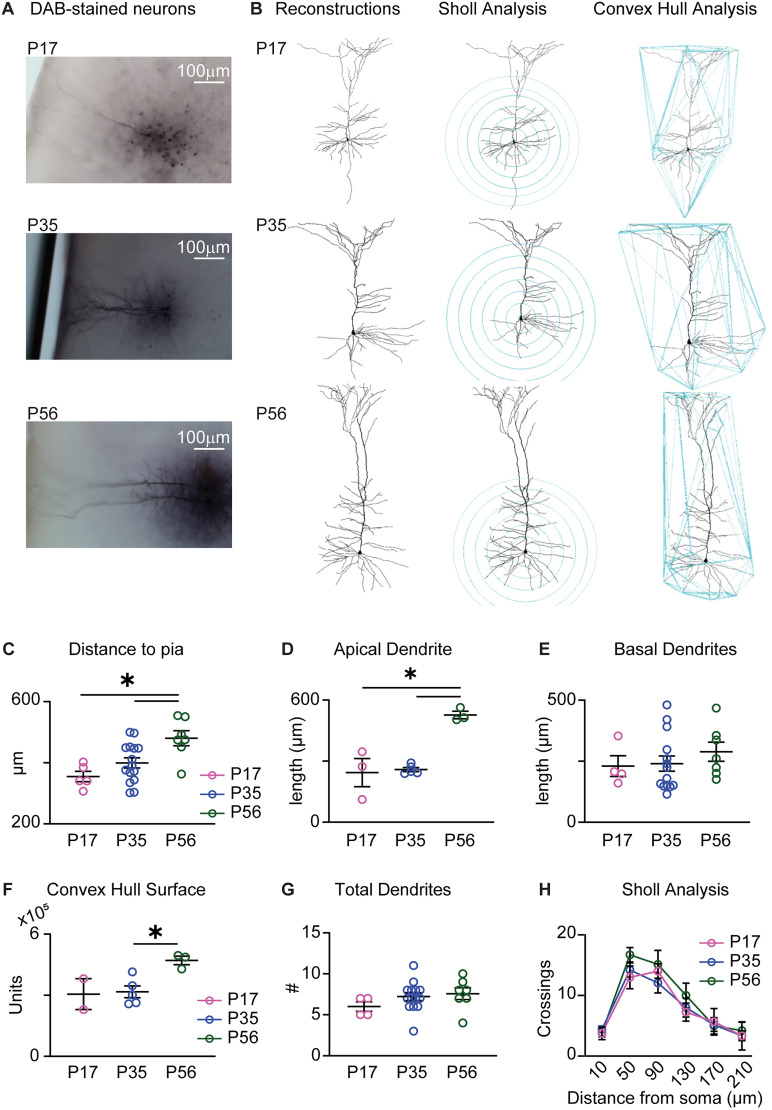
Increased apical dendrite and GC pyramidal neuron surface during postnatal development. ***A***, Example DAB staining for L5 recorded neurons in different age groups taken with a light microscope at 4×. ***B***, Neurolucida reconstructions of dendritic arbors, Sholl analysis, and Convex Hull analysis for reconstructed neurons recorded from P17 (top), P35 (middle), and P56 (bottom). ***C–E***, Plots of distance of soma from the pial surface (***C***), length of apical dendrite (***D***), and length of basal dendrites (***E***). ***F***, Convex hull surface analysis for each age group. ***G***, Total number of dendrites for each age group. ***H***, Sholl analysis on reconstructed neurons by age group. The data points represent average values by age group ± SEM; pink, P17; blue, P35; dark green, P56; asterisks, *p* ≤ 0.05.

Changes in DIR may be related to intrinsic membrane properties or correlate with the complexity of neuronal morphology, which tends to increase during postnatal development (Elston and Fujita, 2014; Ciganok-Huckels et al., 2023). To quantify dendritic morphology, we converted a subset of fluorescently labeled neurons included in the analysis of intrinsic properties to DAB staining. The pyramidal morphology and deep layer location of the cell body had already been verified with fluorescent imaging overlayed with NeuroTrace to identify cortical layers. Slices reprocessed for DAB staining (Fig. 2*A*) facilitated the visualization of processes across visual planes and their reconstruction with Neurolucida software (Fig. 2*B*) while avoiding photobleaching. For each neuron, we quantified the number of dendritic branches, dendritic length, volume of their dendritic arborizations, and dendritic complexity (Fig. 2*B–H*). It should be noted that all neurons included in this morphological analysis showed comparable electrophysiological signature (Fig. 1) and apical dendrites extending tufted arborizations into layer 1, consistent with the typical morphology of layer 5 pyramidal neurons. The average distance of cell bodies from the pial surface and the average length of apical, but not basal, dendrites were significantly increased in GC neurons in P56 mice compared with P17 and P35 suggesting an increase in cortical thickness from P17 to P56 (Fig. 2*C*, somata distance from the pia: P1,7 *n* = 5 neurons; P35, *n* = 15 neurons; P56, *n* = 7 neurons; *F*_(2,24)_ = 6.892, *p* = 0.0043, one-way ANOVA with Tukey's post hoc test: P17 vs P35 *p* = 0.3481; P17 vs P56 *p* = 0.0049; P35 vs P56 *p* = 0.0211; Fig. 2*D*, apical dendrite length: P17 *n* = 3 neurons; P35 *n* = 5 neurons; P56 *n* = 3 neurons; *F*_(2,8)_ = 20.03, *p* = 0.0008; post hoc: P17 vs P35 *p* = 0.9437; P17 vs P56 *p* = 0.0016; P35 vs P56 *p* = 0.0011; Fig. 2*E*, basal dendrites length: P17 *n* = 4 neurons; P35 *n* = 14 neurons; P56 *n* = 7 neurons; *F*_(2,22)_ = 0.5585). Convex hull analysis revealed a greater volume of arborizations at P56 compared with younger ages (Fig. 2*F*, convex hull surface: P17 *n* = 2 neurons; P35 *n* = 5 neurons; P56 *n* = 3 neurons; *F*_(2,7)_ = 5.832, *p* = 0.0323, one-way ANOVA with Tukey's post hoc test: P17 vs P35 *p* = 0.9773; P17 vs P56 *p* = 0.0689; P35 vs P56 *p* = 0.0377). Differently, the total number of dendrites and the dendritic complexity, quantified with Sholl analysis, did not change (Fig. 2*G*, number of dendrites: P17 *n* = 4 neurons; P35 *n* = 14 neurons; P56 *n* = 7 neurons; *F*_(2,22)_ = 1.061, *p* = 0.3630, one-way ANOVA; Fig. 2*H*, Sholl analysis: P17 *n* = 4 neurons; P35 *n* = 14 neurons; P56 *n* = 7 neurons; *F*_(2,22)_ = 0.4696, *p* = 0.9474, two-way RM ANOVA with main effect of age). These data suggest that despite the increase in length of the apical dendrite and the increased volume, the complexity of pyramidal neuron morphology is largely stable across the ages we examined. While an increase in cell surface and apical dendrite length may explain the decrease in DIR at P56, it is inconsistent with the recovery of excitability indicated by the input/output function and maximum firing rate in the absence of changes in rheobase and action potential threshold.

### Developmental decrease in E/I balance onto GC pyramidal neurons

**Figure 3.**
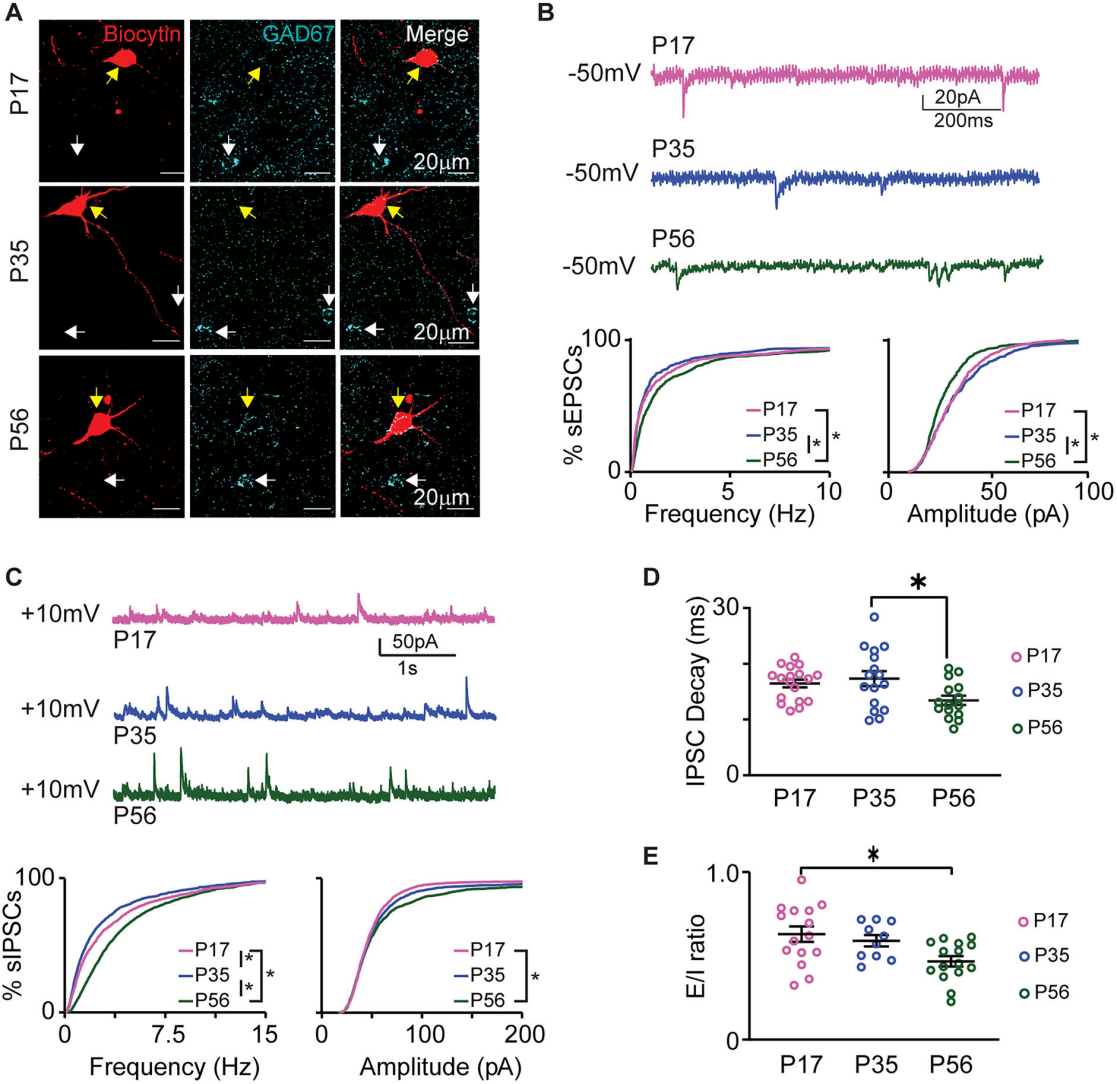
Shift in E/I balance during postnatal development. ***A***, Sample single plane confocal images of biocytin-filled recorded neurons showing lack of colocalization with GAD67. The rows show a sample recorded neuron for each age. Yellow arrows, recorded neurons filled with biocytin (red); white arrows, GAD67^+^ neurons at the same tissue depth. The lack of overlap confirms that recorded neurons are glutamatergic. ***B***, Top, Sample traces of sEPSCs recorded from P17 (pink), P35 (blue), and P56 (green) mice. Bottom, Cumulative distributions of sEPSCs frequency and amplitude by age group. ***C***, Top, Sample traces of sIPSCs recorded from P17 (pink), P35 (blue), and P56 (green) mice. Bottom, Cumulative distributions of sIPSC frequency and amplitude by age group. ***D***, Plot of sIPSC decay time constant by age group. Pink, P17; blue, P35; dark green, P56. ***E***, Ratio of sEPSC and sIPSC charge transfer for each age group. Data are presented as average ± SEM; open circles, single cell data; asterisks, *p* ≤ 0.05.

Previous work from other sensory cortices reported significant changes in synaptic transmission onto pyramidal neurons during postnatal development. While a recent study observed changes in synaptic transmission in GC following taste experience early in life (Schiff et al., 2023), information about developmental changes in synaptic drive onto GC neurons is lacking. To fill this gap in knowledge, we obtained voltage-clamp recordings from GC layer 5 pyramidal neurons in acute slice preparations obtained from mice at ages P17, P35, and P56. For these experiments, we used an internal solution containing Cs_2_SO_4_ and the Na^+^ channel blocker QX314 to stabilize recordings during sustained depolarizations; therefore, recorded neurons could not be identified by their firing pattern. The pyramidal morphology and location of recorded neurons was confirmed post hoc, as was the lack of expression of the inhibitory marker GAD67 (Fig. 3*A*). Spontaneous excitatory and inhibitory postsynaptic currents (sEPSCs and sIPSCs) were recorded by holding neurons at the reversal potential for chloride (see Materials and Methods; Fig. 3*B*) and at the reversal potential for AMPA and NMDA receptor-mediated currents (see Materials and Methods; Fig. 3*C*), respectively.

Analysis of sEPSCs revealed opposite age-dependent changes in frequency and amplitude (Fig. 3*B*, bottom; frequency, left, P17: 2.71 ± 0.49 Hz, *n* = 15 cells from 7 mice; P35: 2.91 ± 0.78 Hz, *n* = 10 cells from 5 mice; P56: 3.07 ± 0.39 Hz, *n* = 15 cells from 10 mice; *H* = 32.556, *p* = 8.5 × 10^−8^; P17 vs P35 *p* > 0.999; P17 vs P56 *p* = 5.566 × 10^−6^; P35 vs P56 *p* = 2.405 × 10^−6^; amplitude, right, P17: 37.89 ± 2.00 pA; P35: 40.82 ± 3.68 pA; P56: 34.32 ± 1.31 pA; *H* = 35.272, *p* = 2.191 × 10^−9^; p17 vs p35 *p* > 0.999; P17 vs P56 *p* = 1.960 × 10^−6^; P35 vs P56 *p* = 8.860 × 10^−7^; Kruskal–Wallis test with Dunn's post hoc test). We did not observe differences in sEPSCs decay time constant or excitatory charge transfer (decay time constant: P17: 6.41 ± 1.08 ms; P35: 5.13 ± 0.51 ms; P56: 7.28 ± 0.703 ms; *F*_(2,35)_ = 1.373, *p* = 0.2667, one-way ANOVA; excitatory charge transfer: P17: 20.71 ± 1.36 pC; P35: 22.83 ± 2.10 pC; P56: 19.64 ± 0.76 pC; *F*_(2,49)_ = 1.604, *p* = 0.212, one-way ANOVA), suggesting that the increase in sEPSCs frequency may be compensated by the decrease in sEPSC amplitude.

When assessing inhibitory synaptic drive (Fig. 3*C*), we observed that the frequency of sIPSCs was transiently reduced from P17 to P35, and then by P56 it increased to become significantly higher than both P17 and P35 (Fig. 3*C*, bottom left, frequency, P17: 3.52 ± 0.41 Hz, *n* = 18 cells from 8 mice; P35: 3.93 ± 0.24 Hz, *n* = 18 cells from 9 mice; P56: 4.52 ± 0.33 Hz, *n* = 16 cells from 8 mice; *H* = 320.718, *p* < 1.0 × 10^−14^; P17 vs P35 *p* = 2.907 × 10^−6^; P17 vs P56 *p* < 1.0 × 10^−14^; P35 vs P56 *p* < 1.0 × 10^−14^ Kruskal–Wallis test with Dunn's post hoc tests), suggesting a transient decrease in inhibitory drive followed by an increase in inhibition by young adulthood. The amplitude of sIPSCs on the other hand showed a significant increase when comparing the youngest age group (P17) to P56 (Fig. 3*C*, bottom right, amplitude, P17: 50.27 ± 3.09 pA; P35: 59.24 ± 4.44 pA; P56: 68.86 ± 8.74 pA; *H* = 14.23, *p* = 8.0 × 10^−4^; P17 vs P35 *p* = 0.136; P17 vs P56 *p* = 5.0 × 10^−4^; P35 vs P56 *p* = 0.205 Kruskal–Wallis test with Dunn's post hoc tests), suggesting an overall increase in inhibitory drive in young adulthood. Analysis of decay kinetics showed a significant reduction in decay time constant selectively between P35 and P56 (Fig. 3*D*, P17: 16.46 ± 0.71 ms; P35: 17.33 ± 1.34 ms; P56: 13.45 ± 0.85 ms; *F*_(2,46)_ = 4.000, *p* = 0.0250, one-way ANOVA with Tukey's post hoc tests: P17 vs P35 *p* = 0.8054; P17 vs P56 *p* = 0.0901; P35 vs P56 *p* = 0.0259). This effect is consistent with previous reports from other brain regions where the decrease in decay time constant was associated with a shift in expression of GABA_A_ receptor subunits on pyramidal neurons (Heinen et al., 2004). The charge transfer of inhibitory synaptic events showed a trend toward increasing from P17 to young adulthood; however, it was not statistically significant across age groups (P17: 38.06 ± 3.55 pC; P35: 38.10 ± 2.66 pC; P56: 45.51 ± 4.60 pC; *F*_(2,49)_ = 1.351, *p* = 0.2686, one-way ANOVA). Overall, both frequency and amplitude of the sIPSCs showed a net increase from P17 to young adulthood.

While on average the excitatory and inhibitory charge transfer was not significantly different, it showed opposite trends, suggesting that the E/I ratio may shift in the developmental window we investigated. We tested this possibility by calculating the ratio of excitatory and inhibitory charge (E/I ratio) for each recorded neuron and compared it across age groups. The E/I ratio was below 1 in all age groups, indicating that the balance between E/I balance favors inhibition in GC. While this parameter was stable between P17 and P35, it decreased significantly by P56 (Fig. 3*E*, P17: 0.62 ± 0.046, *n* = 15 cells from 7 mice; P35: 0.59 ± 0.034, *n* = 10 cells from 5 mice; P56: 0.47 ± 0.030, *n* = 15 cells from 8 mice; *F*_(2,37)_ = 5.156, *p* = 0.011, one-way ANOVA with Tukey's post hoc tests: P17 vs P35 *p* = 0.779; P17 vs P56 *p* = 0.0098; P35 vs P56 *p* = 0.1024), indicating that inhibition becomes even more dominant as neurons reach their mature state.

### Increased PV expression and accumulation of perineuronal nets during postnatal development

**Figure 4.**
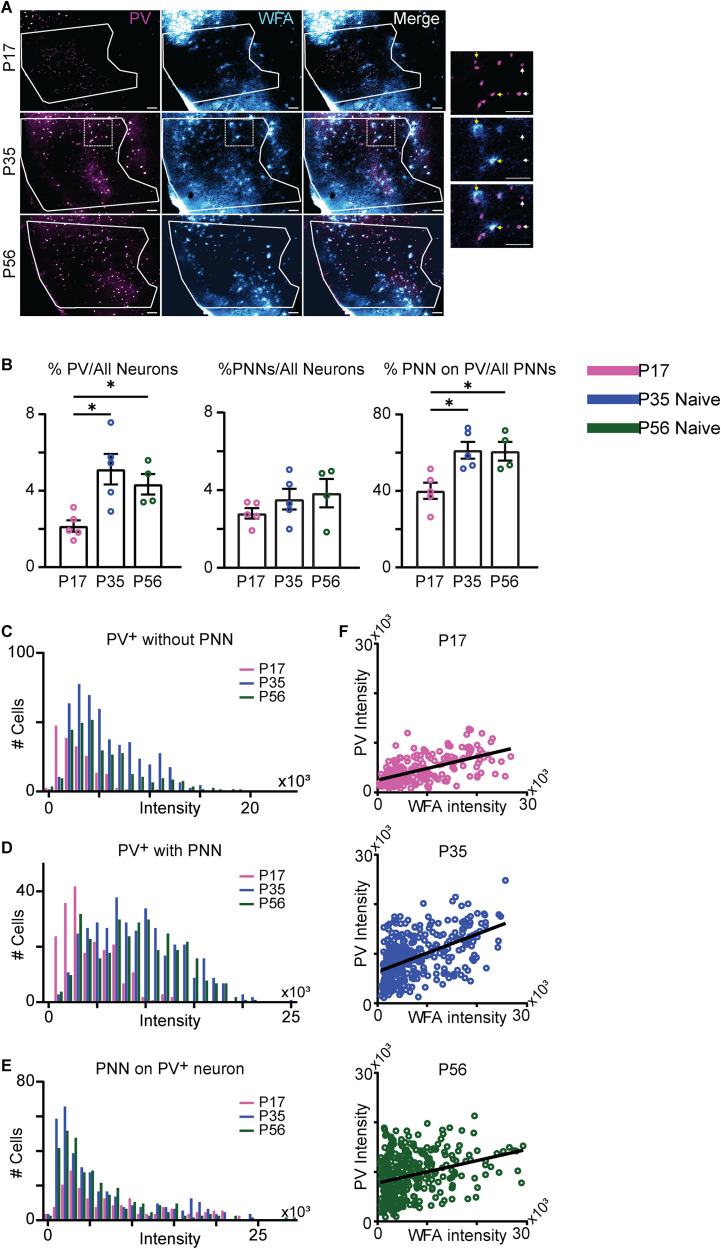
Increased PV expression and accumulation of PNNs during postnatal development. ***A***, Sample images of GC slices processed for immunohistochemistry to label PV (magenta) and WFA to label PNNs (cyan). The region of interest containing GC is outlined in white. Sample images are *z*-stacks with 2 µm step size taken at 10× magnification (scale bar, 100 µm). The rows show PV, WFA, and a merged view for each age. The final column shows a zoomed in image of the boxed region outlined in the P35 sample. White arrows, sample PV^+^ INs without a PNN; yellow arrows, sample PV^+^ INs with a PNN. ***B***, Left, Percentage of PV^+^ INs in GC. Middle, Percentage of GC neurons with a PNN. Right, Percentage of PNNs associated with PV^+^ INs. Open circles, counts by animal. Pink, P17; blue, P35; dark green, P56. ***C***, Distribution of the intensity of the PV signal for PV^+^ INs without a PNN. ***D***, Distribution of the intensity of the PV signal for PV^+^ INs with a PNN. ***E***, Distribution of the intensity of the WFA signal for PNNs associated with PV^+^ INs. ***F***, Correlation of PV intensity and WFA intensity for PV^+^ INs with a PNN. Open circles, normalized fluorescence intensity for single neurons. Solid line, linear regression fit. Pink, P17; blue, P35; dark green, P56; asterisks, *p* ≤ 0.05.

The increase in sIPSC frequency and amplitude as well as the developmental shift of the E/I ratio favoring inhibition suggest a protracted postnatal maturation of inhibitory circuits in the deep layers of GC. Comparable changes have been observed in other cortical regions where they have been associated with PV^+^ INs (Heinen et al., 2004), a neuron type known to undergo several developmental changes in the temporal window we assessed in our experiments (Fagiolini and Hensch, 2000; Chattopadhyaya et al., 2004, 2007; Wu et al., 2012; Tatti et al., 2017). PV^+^ INs make synapses around the perisomatic region of pyramidal neurons (Chattopadhyaya et al., 2004; Kubota et al., 2016), making them the likely source of the majority of sIPSCs we recorded with patch clamp at the soma. Given these premises, we tested the possibility that PV^+^ INs maturation extends postnatally and that their connectivity onto GC layer 5 pyramidal neurons is refined over postnatal development. We compared two well-characterized markers of PV^+^ INs maturation, the expression of PV and the accumulation of PNNs around PV^+^ INs somata (Pizzorusso et al., 2002; Lupori et al., 2023) across age groups. We used immunohistochemistry with an antibody against PV and the histological stain Wisteria Floribunda Agglutinin (WFA) to label PNNs in GC (Fig. 4*A*) and quantified the proportion of GC neurons expressing PV, the overall proportion of PNNs and the proportion of PNNs on PV^+^ INs at P17, P35, and P56.

The proportion of PV^+^ INs increased from P17 to P35 and then was stable to P56 (Fig. 4*B*, left, P17, 2.15 ± 0.30%, *n* = 5 mice; P35, 5.12 ± 0.80%, *n* = 5 mice; P56, 4.34 ± 0.54%, *n* = 4 mice; *F*_(2,11)_ = 7.117, *p* = 0.0104, one-way ANOVA with Dunnett's post hoc tests: P17 vs P35 *p* = 0.007; P17 vs P56 *p* = 0.050). The overall proportion of PNNs in GC did not change across age groups (Fig. 4*B*, middle, P17, 2.81 ± 0.27%, *n* = 5 mice; P35, 3.53 ± 0.53%, *n* = 5 mice; P56, 3.85 ± 0.73%, *n* = 4 mice; *F*_(2,11)_ = 1.077, *p* = 0.3740, one-way ANOVA). However, the proportion of PNNs on PV^+^ INs increased significantly from P17 to P35 and then remained stable (Fig. 4*B*, right: P17, 40.09 ± 4.24%, *n* = 5 mice; P35, 61.35 ± 4.37%, *n* = 5 mice; P56, 60.81 ± 4.89%, *n* = 4 mice; *F*_(2,11)_ = 7.597, *p* = 0.0085, one-way ANOVA with Dunnett's post hoc tests: P17 vs P35 *p* = 0.0096; P17 vs P56 *p* = 0.0158). These results suggest that both PV expression and association of PNNs with PV^+^ INs increase during a protracted period of postnatal development.

Next, we examined the distribution of the intensity of the fluorescent PV signal in PV^+^ INs with and without a PNN during postnatal development and the fluorescence intensity of PNNs labeling on PV^+^ INs. The expression of PV is activity dependent and can be measured as changes in fluorescence intensity (Donato et al., 2013). The intensity of the WFA fluorescent signal informs on the degree of accumulation of a PNN (Slaker et al., 2016; Lupori et al., 2023; Santos-Silva et al., 2024). For all age groups, we sorted PV^+^ INs into two groups, one without an associated PNN and one with a PNN. Neurons were defined as PV^+^ IN if they showed colocalization of the NeuroTrace signal with the PV signal. They were also identified as associated with a PNN if the PV and NeuroTrace signals were surrounded by clustered WFA fluorescence. The PV^+^ IN with PNN included PV neurons with mature (high intensity and accumulation) and immature (low intensity and more diffuse cluster of fluorescence) around the somata (Fig. 4*A*). PV^+^ INs without a PNN were defined as neurons showing colocalization of the NeuroTrace and PV signals whose soma was not surrounded by WFA fluorescence. The fluorescence signal was quantified as distribution of intensity versus cell number for each age group (Fig. 4*C–E*). For statistical comparisons across slices and animals, the background fluorescence intensity (measured as the average intensity of the entire GC for each imaging channel) was subtracted from the corresponding channel fluorescence intensity for each identified PV and WFA ROI.

In PV^+^ INs without a PNN, the histogram of PV signal intensity showed an increase in PV fluorescence intensity across age groups (Fig. 4*C*; PV^+^ INs without PNN: P17 *n* = 184 cells from 5 mice; P35 *n* = 503 cells from 5 mice; P56 *n* = 317 cells from 4 mice; *H* = 125.3, *p* < 1.0 × 10^−10^; P17 vs P35 *p* < 1.0 × 10^−10^; P17 vs P56 *p* < 1.0 × 10^−10^; P35 vs P56 *p* = 0.3225; Kruskal–Wallis with Dunn's post hoc test). These results are consistent with a developmental increase in PV expression, which has also been observed in other cortical regions (Gonchar et al., 2007; Tatti et al., 2017).

Analysis of the distribution of PV signal intensity for PV^+^ INs with a PNN showed an increase in intensity between P17 and P35 and no change between P35 and P56 (Fig. 4*D*; PV^+^ with PNN: P17 *n* = 208 cells from 5 mice; P35 *n* = 382 cells from 5 mice; P56 *n* = 343 cells from 4 mice; *H* = 192.2, *p* < 1.0 × 10^−10^; P17 vs P35 *p* < 1.0 × 10^−10^; P17 vs P56 *p* < 1.0 × 10^−10^; P35 vs P56 *p* > 0.9999; Kruskal–Wallis with Dunn's post hoc test), suggesting that after P35 the number of PV^+^ INs and their association with a PNN reached adult levels and that once PV^+^ INs become associated with a PNN the PV expression was stable.

We quantified the fluorescence intensity of WFA labeling of PNNs associated with PV^+^ INs as a measure of PNN accumulation specifically on PV^+^ INs. There was an increase in the distribution of WFA fluorescence intensity between P17 and P35, suggesting age-dependent aggregation of PNNs (Fig. 4*E*; P17 *n* = 5 mice; P35 *n* = 5 mice; P56 *n* = 4 mice; *H* = 21.35, *p* = 0.2.314 × 10^−5^; P17 vs P35 *p* = 3.157 × 10^5^; P17 vs P56 *p* = 3.264 × 10^−4^; P35 vs P56 *p* > 0.999; Kruskal–Wallis with Dunn's post hoc test), suggesting that the developmental increase in WFA fluorescence depends on an increase in the number of PV^+^ INs associated with a PNN, rather than an increase in average fluorescence intensity.

Finally, we asked whether the intensity of the normalized PV and WFA signals covaried for each dual-labeled neuron within each age group. To do that, PV fluorescence intensity normalized to the corresponding slice NeuroTrace signal for each cell was plotted against each cell's normalized WFA signal (Fig. 4*F*) and their correlation quantified with a Spearman correlation test. The PV and WFA fluorescence signals were significantly correlated in each age group (P17 *r* = 0.556, *p* < 0.001; P35 *r* = 0.514, *p* < 0.001; P56 *r* = 0.307, *p* < 0.001, Spearman ranked correlation). The correlation did not increase with age, but the PV intensity signal increased, supporting the interpretation that PV expression increases during postnatal development and PNNs accumulate on PV^+^ INs reaching their mature state by P56.

### Refinement of PV^+^ INs—pyramidal neurons connectivity during postnatal development

**Figure 5.**
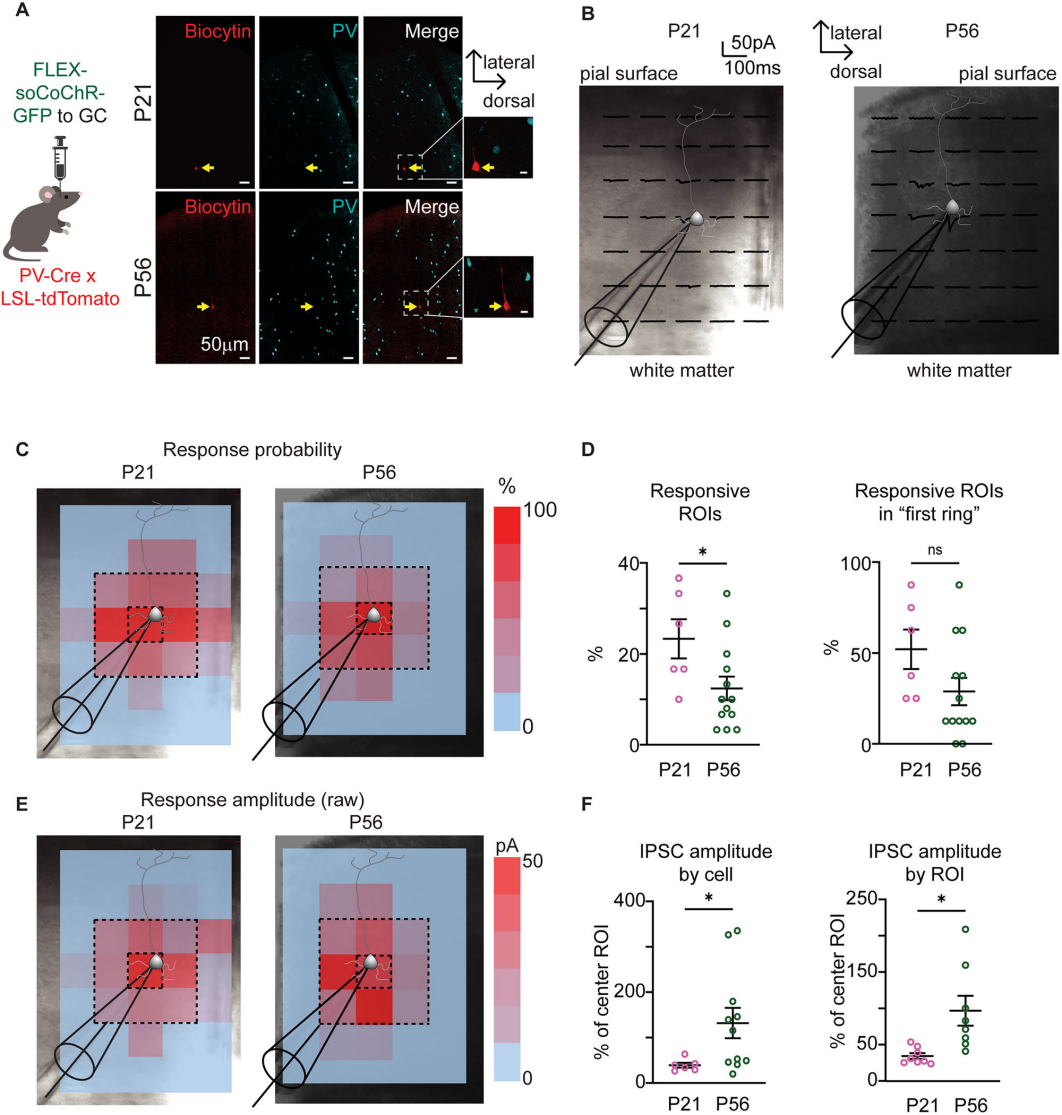
Refinement of PV connectivity and IPSC amplitude during postnatal development. ***A***, Left, Diagram of experimental approach. Right, Sample images of recorded neuron and ChR2-expressing PV neurons. Red, biocytin; cyan, ChR2-PV. Scale bar, 50 µm. Insets, zoomed in images of biocytin-filled neurons. Scale bar: 10 µm. ***B***, Sample responses in 100 µm × 100 µm ROIs around a recorded neuron at P21 (see also Extended Data Fig. 5-1 for comparison of P17 and P21 neuronal excitability) and P56. Tip of the recording electrode diagram: position of recorded neuron's soma, represented by a diagram. ***C***, Sample heat map of the probability of evoking a response in each ROI. Tip of the recording electrode diagram: position of recorded neuron's soma. ***D***, Left, Plot of the percentage of stimulated ROIs (30 ROIs total) which evoked an IPSC. Open circles, values for each recorded neuron; black line, average value. Right, Plot of the percentage of stimulated ROIs within the “first ring” (8 ROIs total), which evoked an IPSC. Open circles, values for each recorded neuron; black line: average value. Pink, P21; dark green, P56. ***E***, Sample heat map of IPSC amplitude in each ROI. ***F***, Left, Plot of IPSC amplitude for stimulated ROIs in the “first ring” presented as % of the amplitude of the IPSC evoked by stimulating the ROI containing the soma of the recorded neuron. Normalized IPSC amplitudes are presented as average for each cell. Open circles, values for each recorded neuron. Right, Plot of evoked IPSC amplitude as % of the IPSC evoked by stimulation of the ROI containing the recorded neuron's soma. Open circles, values for each ROI; pink, P21; dark green, P56. Data are presented as average ± SEM; asterisks, *p* ≤ 0.05.

The developmental increase in inhibitory synaptic drive shown in Figure 3 is likely dependent on changes in perisomatic inhibition, as sIPSCs originating from synapses around the soma of pyramidal neurons are better represented in whole-cell patch-clamp recordings. The increased sIPSC frequency together with the developmental regulation of markers of PV^+^ INs maturation suggests the possibility that the connectivity of PV^+^ INs onto pyramidal neurons undergoes refinement from P17 to young adulthood. To assess PV^+^ IN connectivity onto GC pyramidal neurons, we took advantage of CRACM (Petreanu et al., 2007). We injected an AAV construct carrying a soma-restricted channelrhodopsin sequence (Flex-soCoChr-GFP AAV; Shemesh et al., 2017; Fig. 5*A*) into GC of PV-cre;Ai14 mice using stereotaxic surgery. This approach restricts the expression of the soma-restricted opsin to PV^+^ INs of the injected hemisphere. One week after surgery, we obtained coronal slices containing GC and performed patch-clamp recordings from layer 5 pyramidal neurons (Fig. 5*B*). After establishing the voltage-clamp configuration, we used patterned light stimulation with a digital micromirror device (Polygon, Mightex) to shine blue LED light (473 nm) onto spatially restricted regions of interest (30 ROIs ∼100 × 100 µm each) surrounding recorded neurons (∼500 µm dorsal to ventral and 700 µm from the bottom of layer 1 to the white matter/corpus callosum). We then determined the light-evoked response (or lack thereof) for each ROI (Fig. 5*C*; see Materials and Methods). The number of PV^+^ INs at P17 was too low to drive sufficient expression of channelrhodopsin to effectively evoke synaptic responses onto pyramidal neurons in this age group. After verifying that intrinsic properties were comparable between P17 and P21 (Extended Data Fig. 5-1), we assessed PV^+^ IN connectivity onto layer 5 pyramidal neurons at P21 (viral injection at P14) and compared it to that quantified at P56 (viral injection at P49). Recorded neurons were determined to be putatively pyramidal by their morphology, action potential properties, and absence of colocalization of biocytin with the tdTomato signal. The location and morphology were then confirmed post hoc with fluorescence immunohistochemistry.

10.1523/ENEURO.0329-25.2026.f5-1 Figure 5-1 **Postnatal developmental changes in intrinsic excitability of GC layer 5 pyramidal neurons.** **A.** Average input/output function for neurons recorded at P17 (pink), P21 (black) and P35 (blue). Data presented as mean ± SEM. **B.** Left. Rheobase for each recorded neuron in each age group. Right. Action potential (AP) threshold for each recorded neuron by age group. **C.** Left. Maximum firing frequency for all recorded neurons plotted by age group. Right. Dynamic input resistance (DIR) quantified as the slope of the input/output function for steps below rheobase. Data are reported as average ± SEM. Pink: P17; black: P21; blue: P35. The data for P17 and P35 are the same as in Fig. 1. Asterisks: p ≤ 0.05. Download Figure 5-1, TIF file.

To be included in the analysis, neurons had to respond with an IPSC to light stimulation in at least 1 ROI. For each recorded neuron, we first analyzed the percentage of ROIs that, when stimulated, evoked a detectable IPSC (out of 30 ROIs/pyramidal neuron). Heat maps show the probability of evoking an IPSC by stimulation in each defined ROI within the grid (Fig. 5*C*). This parameter was averaged across each corresponding ROIs for all neurons and compared by age groups. We found that the number of ROIs that when stimulated evoked an IPSC in neurons recorded in slices from P56 mice was smaller compared with P21 (Fig. 5*D*, left; P21: *n* = 6 cells from 3 mice, IPSCs evoked in 7.0 ± 1.29 out of 30 ROIs; P56: *n* = 13 cells from 6 mice, IPSCs evoked in 3.69 ± 0.91 out of 30 ROIs; *U* = 15, *p* = 0.0331, Mann–Whitney *U* test). We then restricted the analysis to the ring of activated ROIs immediately surrounding the soma of the recorded neuron and compared responsiveness to activation of each ROI in this “first ring” (eight total ROIs, indicated by the dashed line in Fig. 5*D*). We observed a trend toward reduced connectivity at P56 that did not reach statistical significance (Fig. 5*D*, right, P17: 4.17 ± 0.87 out of 8 ROIs, P56: 2.3 ± 0.9 out of 8 ROIs; *U* = 18.5, *p* = 0.0659, Mann–Whitney *U* test). Thus, at P21 pyramidal neurons receive input from PV^+^ IN whose somata are distributed over a wider spatial map compared with P56. These results point to a refinement of PV^+^ IN circuits during the developmental window under study.

The refinement of connectivity of PV^+^ INs at P56 correlates with the postnatal time window showing an increase in sIPSCs frequency and amplitude (Fig. 3). We, therefore, tested the possibility that the amplitude of evoked IPSCs may be larger at P56 compared with P21. This analysis was restricted to IPSCs evoked by stimulating ROIs in the “first ring” surrounding the recorded neuron indicated by dashed lines in Figure 5*E*, as they showed the highest probability of eliciting an IPSC in both age groups. Heat maps of IPSC amplitudes (Fig. 5*E*) show differences in the raw IPSC amplitude of IPSCs activated by stimulation of the ROIs. To compare IPSC amplitudes across neurons, we normalized data in two ways. In the plots in Figure 5*F* we report IPSC amplitude normalized to the amplitude of the IPSC evoked by stimulating the center ROI for each recorded neuron. This approach accounts for differences in virus expression and light stimulation across preparations. IPSCs recorded in P56 mice were significantly larger compared with IPSCs from slices of P21 mice (Fig. 5*F*, left, P21 *n* = 6 cells from 3 mice; P56 *n* = 13 cells from 6 mice; *U* = 11, *p* = 0.0273, Mann–Whitney *U* test). At P21 the amplitude of the IPSCs evoked by stimulating the “first ring” ROIs was smaller compared with that of IPSC evoked by stimulating the central ROI. Differently, at P56 the amplitude of IPSCs evoked by stimulating the “first ring” of ROIs was larger compared with that evoked by focusing the light stimulus to the central ROI. To further confirm this observation, we plotted the normalized IPSCs evoked by stimulation of each ROI to test for potential confounds due to the location of the stimulation. At P56 more activated ROIs evoked IPSCs with amplitude larger than the IPSC evoked by stimulation of the central ROI (Fig. 5*F*, right, IPSC amplitude by ROI, *n* = 8 ROIs for each age group; *U* = 3, *p* = 0.0011, Mann–Whitney *U* test), confirming the age-dependent increase in IPSC amplitude evoked by stimulating PV^+^ IN somata located in the vicinity of the perisomatic region of recorded pyramidal neurons. These results indicate that at P21 layer 5 pyramidal neurons in GC receive inhibitory input from PV^+^ INs distributed over a larger surrounding area, while at P56 they receive input only from PV^+^ IN immediately surrounding the pyramidal neuron somata. Over the same developmental window, the amplitude of PV^+^ IN IPSCs increases, suggesting a more powerful and localized inhibitory control over pyramidal neurons’ excitability. This effect may explain, at least in part, the increase in inhibitory drive shown and with the shift in E/I ratio toward inhibition in Figure 3.

## Discussion

Our results show that the developmental window from P17 to P56, a critical period for the development of taste preference (Schiff et al., 2023) and a time of refinement of gustatory circuits (Bigiani et al., 2002; Ohtubo et al., 2012), is characterized by a decrease in the excitability of pyramidal neurons in layer 5 of GC. This effect is associated with subtle changes in intrinsic membrane properties, with a shift in the E/I balance of overall synaptic drive toward inhibition and a refinement in connectivity and synaptic strength of inhibitory circuits mediated by PV^+^ INs.

The transient decrease in intrinsic excitability of pyramidal neurons in the fifth postnatal week was characterized by a flattening of the input/output function between P17 and P35. Similar effects were recently reported in mouse primary visual cortex (V1) at the transition from eye opening (P14) to P28, the peak of the critical period for ocular dominance plasticity (Ciganok-Huckels et al., 2023), suggesting that this may be a common feature of sensory circuits. In V1, the shift in input/output function is permanent (Ciganok-Huckels et al., 2023), while in GC, the decrease we observed was transient, as the input/output function of pyramidal neurons at P56 was comparable to that of P17 mice. The decrease in excitability at P35 was not accompanied by changes in rheobase and action potential threshold as reported for V1 (Ciganok-Huckels et al., 2023) and prefrontal cortex (Kroon et al., 2019). GC layer 5 pyramidal neurons showed a decrease in input resistance between P17 and P56, suggesting decreased intrinsic excitability despite the recovery of input/output function. The decrease in input resistance may be explained by the increased cell surface. An increase in pyramidal neurons size was previously observed in the prefrontal cortex, although it occurred much earlier in development (Kroon et al., 2019). Our findings in GC also differ from the morphological changes reported for V1, where basal dendrites, but not apical dendrites, increased in size (Ciganok-Huckels et al., 2023), suggesting region-specific developmental changes in the laminar distribution of inputs onto pyramidal neurons. Taken together, our results are in line with findings from other cortical regions in reporting an overall decrease in deep layer pyramidal neurons’ intrinsic excitability and changes in pyramidal neurons’ morphology between juvenile and young adult mice during postnatal development and suggest the engagement of region-specific mechanisms.

The observed changes in synaptic drive were delayed compared with the shift in input/output function: sEPSC and sIPSC amplitude and frequency were comparable between P17 and P35, while both parameters were significantly different at P56, suggesting that the postnatal development of synaptic function in GC extends beyond the 5th postnatal week, after critical periods for other sensory cortices have closed (Antonini et al., 1999; Hensch, 2004; Southwell et al., 2010). The developmental decrease in sEPSC amplitude was accompanied by a small but significant increase in sEPSC frequency. This effect is comparable to previous reports from V1 (Desai et al., 2002; Tatti et al., 2017), although the changes in GC occur later in development.

Inhibitory synaptic drive was overall increased as indicated by higher sIPSC frequency and amplitude. There was also a decrease in the decay kinetics from P35 to P56 which may partially compensate for the increase in amplitude and frequency, as the overall charge transfer for inhibitory events showed a trend toward larger values. When assessing the E/I ratio of change transfer for each recorded neuron, we observed a significant shift toward inhibition. This effect, together with the decrease in input resistance, renders GC layer 5 neurons less excitable in young adulthood compared with the earlier age groups and points to a powerful inhibitory control of GC output.

In rodent V1, a decrease in sIPSC kinetics in the presence of increased amplitude is associated with a switch from a prevalence of GABA_A_ receptors containing the α3 subunit to GABA_A_ receptors containing the α1 subunit, a process that is complete by the third postnatal week and is an important mechanism regulating the process of maturation of inhibitory circuits and the opening of the critical period for ocular dominance plasticity (Fagiolini and Hensch, 2000; Heinen et al., 2004). The acceleration of sIPSC decay kinetics in GC occurs between the fifth and seventh postnatal week, raising the possibility that the maturation of inhibitory synapses in this brain region extends into adulthood. In addition to changes in sIPSC amplitude and kinetics, we observed a significant increase in sIPSC frequency by P56, which is typically associated with modulation of presynaptic release (Zucker and Regehr, 2002).

The increase in apical dendrite length together with the augmented cell surface are expected to increase membrane capacitance, an effect that could explain the decrease in input resistance observed at P56 (Marty and Neher, 1995). In addition, a longer dendrite may explain the decreased sEPSC amplitude (Segev and London, 2000). However, increased cell capacitance and decreased input resistance are not consistent with the observation that the P56 input/output function is restored to P17 levels in the absence of changes in rheobase and action potential threshold across age groups. Furthermore, the apical dendrite elongation does not explain the increase in sIPSCs amplitude, nor the decrease in sIPSC decay time constant. An increase in cell surface and longer dendritic processes predict the opposite effects in the absence of other factors such as changes in connectivity, recruitment of active conductance, or plasticity (Segev and London, 2000; Chklovskii et al., 2004).

In patch-clamp recordings from pyramidal neurons somata, the majority of sIPSCs are generated by perisomatic inputs, which are primarily from PV^+^ IN synapses. In sensory cortices, this population of inhibitory neurons undergoes extended postnatal maturation. The number of neurons expressing PV increases during the postnatal period (Gonchar et al., 2007; Tatti et al., 2017) and changes in PV expression are associated with learning (Donato et al., 2013) and experience-dependent plasticity (Chattopadhyaya et al., 2004; Southwell et al., 2010; Schiff et al., 2023). In mouse V1, the expression of PV increases after the second postnatal week (Baho et al., 2019), and PV^+^ INs become associated with PNNs whose accumulation reaches stable levels around the end of the fifth postnatal week, when the critical windows for experience-dependent plasticity closes (Pizzorusso et al., 2002; Berardi et al., 2003; Sorg et al., 2016). In GC, our data show that the expression of PV and accumulation of PNNs on PV^+^ INs increased between P17 and P35, similar to previous reports in other cortices (Lupori et al., 2023). Interestingly, our data in Figures 3 and 5 show that in GC, the accumulation of PNNs around PV^+^ INs is complete by P35, before the increase in inhibitory synaptic drive, providing additional support for the interpretation that in this cortical region the maturation of synaptic transmission extends over a longer postnatal time window compared with other sensory cortices (Lo et al., 2017; Tatti et al., 2017).

Analysis of the connectivity of PV^+^ INs onto layer 5 pyramidal neurons in GC highlights a process of refinement characterized by restriction of connectivity to the perisomatic region, suggesting pruning of connections from PV^+^ INs located further away from the pyramidal neurons’ somata and increase in synaptic efficacy of inputs from PV^+^ IN located close to pyramidal neuron's somata from the third postnatal week to P56. Previous work in organotypic cultures of V1 identified a postnatal increase in GABA with increased pruning of PV^+^ INs synapses leading to a restriction of PV^+^ INs innervation around the perisomatic region of pyramidal neurons (Chattopadhyaya et al., 2007; Wu et al., 2012). As changes in frequency of spontaneous synaptic currents are associated with changes in neurotransmitter release (Zucker and Regehr, 2002), it is tempting to speculate that the developmental increase in sIPSCs frequency and amplitude relies on a combination of increase in GABA release and refinement of PV^+^ IN connectivity around the perisomatic region of GC layer 5 pyramidal neurons.

The picture emerging from our analysis points to an extended postnatal process of maturation of cortical circuits for taste that straddles the developmental window between the third and seventh postnatal week. This process involves intrinsic and synaptic changes that decrease layer 5 pyramidal neurons excitability and shift in E/I balance of synaptic charge toward inhibition. In parallel, the connectivity of inhibitory circuits mediated by PV^+^ INs are refined to focus their influence on the perisomatic region of layer 5 pyramidal neurons, possibly modulating the output of GC. The timeline of GC maturation overlaps with a developmental window during which experience with tastants has a lasting influence on taste preference that persists into adulthood (Schiff et al., 2023). While this study reports the developmental changes of intrinsic and synaptic inputs occurring over the postnatal window from P17 to P56, future studies are needed to determine whether these changes are independent or coregulated.

Mice begin their transition toward eating solid food around P12–P14 and by P17–P21 they become fully independent eaters (Bechard and Mason, 2010; Bailoo et al., 2020). In laboratory settings mice are typically separated from their mother by P21–P28, in our study at P21, which is considered the time of weaning. The transition to independent feeding marks a highly plastic time in the development of taste processing that can influence taste preferences later in life in humans (Mennella and Beauchamp, 2002; Trabulsi and Mennella, 2012) and mice (Schiff et al., 2023). A recent study in mice revealed that early experience with taste influences the maturation of inhibitory circuits mediated by PV^+^ INs in GC (Schiff et al., 2023). The results we present here indicate that during the period of transition to independent feeding, when mice experience tastes different from their mother's milk, there is extensive refinement of GC pyramidal neurons’ excitability and PV^+^ INs circuits. This period of maturation extends into young adulthood.

When viewed in the context of previous work on postnatal maturation of cortical circuits (Micheva and Beaulieu, 1995; Fagiolini and Hensch, 2000; Chattopadhyaya et al., 2004; Gogolla et al., 2014; Lo et al., 2017; Tatti et al., 2017; Takesian et al., 2018; Ciganok-Huckels et al., 2023; Schiff et al., 2023), our study highlights common features, supporting the interpretation that these may be general mechanisms for the refinement of cortical circuit organization. The events underlying the maturation of cortical circuits appear to occur over region-specific timelines, with GC circuits refinement extending over a longer postnatal window compared with somatosensory, auditory, and visual cortical circuits (Micheva and Beaulieu, 1995; Lo et al., 2017; Tatti et al., 2017; Takesian et al., 2018; Ciganok-Huckels et al., 2023). This feature of GC circuits may depend on the need for extended experience-dependent plasticity for taste to allow for the acquisition of the identity of safe and nutritious food while foraging, a process that engages other sensory and motor systems to find nutrients.

## Data Availability

All data needed to evaluate the conclusions in the paper are included in the manuscript.

## Synthesis

Reviewing Editor: Arvind Kumar, KTH Royal Institute of Technology Decisions are customarily a result of the Reviewing Editor and the peer reviewers coming together and discussing their recommendations until a consensus is reached. When revisions are invited, a fact-based synthesis statement explaining their decision and outlining what is needed to prepare a revision will be listed below. The following reviewer(s) agreed to reveal their identity: Björn Kampa, Donald Katz.

Synthesis

Your manuscript was reviewed by two reviewers. Both reviewers, as you will read in their comments, agree that the work is solid and addresses an important problem. The results are novel and give new insights into the development of the brain circuitry. However, they have raised several concerns that need to be carefully addressed. One of the reviewers argued that GC maturation may not be slower as authors conclude. Given the variability of results the second reviewer has made several suggestions to improve the presentation of results and clarify the analysis and methods.

The full comments from the reviewers are below. Please provide a point-by-point reply to these comments.

Reviewer #1

The authors investigated potential developmental changes in the gustatory cortex (GC) of mice before during and after weaning. The GC was prepared from C57BL/6 mice at P17, P35, and P56 developmental window from pre-weaning to young adulthood.

Since the diet changes during this period, it is expected that the GC also undergoes changes that should be observable in electrophysiological and morphological measurements in ex vivo brain slices. The authors concentrated their measurements on pyramidal neurons in the infragranular deep layers, their intrinsic excitability and synaptic inputs. They found a decreased input/output relationship in P35 mice just after weaning, which was determined in a reduced number of evoked action potentials (AP) during current step injections. However, further measurements of AP threshold or rheobase did not lead to similar results, neither did the input resistance. Synaptic inputs underwent a continuous change with age by decreasing the excitation to inhibition balance. This could be further confirmed by an increase in the number of inhibitory PV positive cells in older mice, also mediating more and larger inhibitory inputs to neighbouring pyramidal neurons.

The study is based on a solid methodology applying state-of-the-art electrophysiology and morphology measurements in brain slices. The data is convincing with a few open points that should be addressed. The overall conclusion, that the developmental changes of the GC occur after weaning and continue into adulthood are backed up by their data. However, alternative explanations should be considered as well.

Major Comments

The authors state that "GC circuits maturation extending over a longer postnatal window compared to somatosensory, auditory and visual cortical circuits". However, weaning can be a fast process. Therefore, developmental changes in GC might also occur on a fast time scale or only in a brief window. Did the recordings occur within a day of the weaning date? Or is there any temporal difference of the measured effects and the time since weaning? Otherwise, it might be possible that weaning induced plastic changes could be missed and that the observed changes are long-term adaptations during adulthood. These could also further change by a change of diet for example to high-fat or high-calorie diet.

The authors did not observe changes in intrinsic properties like AP threshold, rheobase or input resistance to be in line with the reduced I/F curve in P35 mice. However, the AP threshold (in Fig 1) does show a trend to be higher in P35 compared to P17 and P56. This would be in line with the reduced input/output function shown in Fig 1C. To better interpret the AP threshold, it would be important to also show the resting membrane potential and the difference between the two measurements. Was there any difference in resting membrane potential between these age ranges?

Minor comments

Why did the authors focus on pyramidal neurons in deep infragranular layers, and how did they record from infragranular layers in all three divisions of GC (granular, dysgranular and agranular)?

Which electrophysiology recording system was used? Together with which software? Was series resistance compensation done by hand or automatically?

Mice of both genders were used, but was there any sex difference in the results?

Line 83: What is the sensitive window for the development of sweet taste preference.

Line 277, What was compared in two-way RM ANOVA (with main effect of age). Was gender also included as factor? It might be better to use linear mixed effect models for statistical testing to exclude effects of individual mice or gender

Fig 2: DAB staining is difficult to see

Fig 3a shows the same as Fig1a, just with a different subset. It might be better to show all recorded neurons in a supplementary figure.

Were recordings done in consecutive order (first -50mV then +10 mV)? Did order have an effect on recoding result or quality?

Line 341: better explain what is shown here. What are the medians of the frequency or amplitude?

Figure 5: Only 6 cells out of 3 mice recorded at p21. To be compared with p56. Unclear why p35 has not been used?

Reviewer #2

This is a well-done inquiry into developmental changes in gustatory cortical circuitry, showing increases in the strength of PV inhibition onto pyramidal cells between P17 and P56. The authors' most central effect (Figure 3H) is very cool, and itself well worthy of publication. I also like the scholarliness of the comparisons to visual cortical development. While I have many suggestions on how to improve the manuscript, all will be easily handled by the authors, and none dampen my enthusiasm for the work. Those suggestions follow.

The effects are intriguingly but annoyingly varied. In one case, a P17-P35 difference resets by P56, but in other cases it doesn't, and in still other cases change only occurs after P35. Again, this is fascinating, and it also really drives home the authors' point about something happening in the general vicinity of P35 (more on that below), but it is NOT clear that the effects being observed are related to one another. The authors should either provide a conceptual framework whereby the diverse timings of effects can be thought of as part of a single developmental process (e.g., explaining why an earlier change might be thought of as leading to a later change) or be a little more circumspect in their prose (e.g., explicitly discussing the possibility that independent changes are happening at different times).

Given that mouse pup weaning happens in the 3rd week of life, I'm a bit confused as to why the authors didn't test closer to that date. While the authors' data definitely seem to suggest nonlinearities of change, in that things are stable across 2 out of 3 time points and "suddenly" change across one time point and the 3rd, it is not at all clear that these changes are tightly linked to weaning. This particularly true of the changes that occur after P35. I suggest that the authors lean away from organizing their Intro around weaning, a change that would have the added benefit of making the analogy with visual development more compelling.

Relatedly, I see little evidence for gradual changes in the authors' data. Given their sampling, it looks as if for virtually every effect, the data are stable across 2 of 3 time points and different for the 3rd. The authors should either remove language like "progressive decrease" (Line 288), which suggests similar amounts of change between P17 and P35 and between P35 and P56, or else do a better job of motivating that language (by, e.g., performing an analysis that shows that change to be truly gradual).

In Lines 321-325, the authors suggest that the lengthening of the apical dendrite cannot explain the effects described in Figures 1 &2. I believe them, but it would be a good idea to provide some sort of references to work (theoretical work, even) that makes it clear what physiological changes do and don't follow from lengthening of the apical dendrite.

I missed a technical description of how the authors "sorted PV+ 407 INs into 2 groups, one without an associated PNN and one with a PNN." Particularly given my suspicion that some PNNs are less clear than others, the authors should provide a thorough explanation of this analysis (my apologies if they did so and I just missed it).

I have some concerns about the analysis summarized in Figure 4F. I assume that the histology varied in quality (i.e., in general brightness) from mouse to mouse and even slice to slice, and this general variability could cause an artifactual correlation between the brightness of two imaged variables. It would be valuable to demonstrate that this confound did not impact results, either by: 1) adding in a 3rd, unrelated variable and showing that it was uncorrelated with the other two; or 2) doing a within-slice analysis showing that even with any between variability nullified the effect remains.

It is not clear to me how the Figure 5D-F results, which show reductions of connectivity with age, are properly interpreted to suggest that "PV+ INs input onto GC pyramidal neurons becomes more spatially restricted over the course 476 of postnatal development" (Lines 474-477). The authors need to either more clearly explain or remove that claim.

The writing could still use some work. A non-exhaustive list of the issues that I observed includes:

1) Repeated redundancies involving the word "dynamic." As the definition of the word "dynamic" is "changing," expressions like "dynamic changes" means "changing changes." Even expressions such as "dynamic circuit refinement" (Lines 81-82) are subtly redundant.

2) The authors should choose to refer either to "sensitive" and "critical" periods, and not switch between those terms (unless they explicitly explain why they are doing so).

3) Lengthy descriptions of non-significant effects (e.g., Lines 363-366) should be omitted.

4) The authors start a lot of sentences with a single word, followed by a comma, and followed by the rest of the sentence. While commonly used, this is an often awkward and often inappropriate construction, and its use should be minimized. Most notably, the authors should remove the two paragraphs in a row that start with "Finally,..." (Lines 428 and 436).

5) There are a number of agrammaticisms.

Finally, I have quite a few small Figure issues:

- In general, panels in some figures read reliably increasing in rows from left to right, whereas in other figures, panel ordering suddenly switches to being columnar, from top to bottom. This is very confusing, and should be changed.

- For Figure 1D, I'd like x-axis labels despite the colors (which refer to a different panel). Also, I recommend removing 1E-G, which show nothing, so that you can make 1D larger.

- Figure 2D &E could be combined, again allowing the authors to avoid presenting entire panels that show no effects.

- Figure 3A seems to show what was already shown in Figure 1A. It should be removed, because the reader doesn't need to be convinced again that pyramidal cells can be identified that aren't interneurons. Furthermore, the effect in Figure 3D ("opposite age-dependent changes in frequency and amplitude") is non-compelling as presented.

- Figure 4C is also unclear. I assume that the effect to which the authors are referring in Lines 410-411 has to do with how far to the right the cumulative sum plots go for the different samples. If so, the authors need to very explicitly point out that this is the nature of the effect. A better idea, however, would be to actually present the data as distributions (i.e., frequency rather than cumulative frequency in the y-axis).

## Author Response

Dear Editors, We are grateful that our manuscript is considered potentially suitable for publication in eNeuro. We carefully considered the reviewers' feedback and revised the manuscript text and figures to address each concern. Specifically, we re-wrote portions of the abstract, introduction, methods, results and discussion to clarify the study goals, experimental approach and data interpretation. We have edited the figures to simplify them as suggested. Where appropriate, negative results were removed from the figure and included in the text. A supplementary figure was added to demonstrate that the excitability of neurons at P21 matches that of neurons recorded at P17. Cumulative distributions in Fig. 4 were substituted with histograms as suggested by the reviewers. A point-by-point response to the comments follows below.

Reviewer #1 Major Comments The authors state that "GC circuits maturation extending over a longer postnatal window compared to somatosensory, auditory and visual cortical circuits". However, weaning can be a fast process. Therefore, developmental changes in GC might also occur on a fast time scale or only in a brief window. Did the recordings occur within a day of the weaning date? Or is there any temporal difference of the measured effects and the time since weaning? Otherwise, it might be possible that weaning induced plastic changes could be missed and that the observed changes are long-term adaptations during adulthood. These could also further change by a change of diet for example to high-fat or high-calorie diet.

We thank the reviewer for the comment. We have edited the text in the Discussion to address this point (lines 657-669). "Mice begin their transition toward eating solid food around P12-P14 and by P17-P21 they become fully independent eaters (Bechard and Mason, 2010; Bailoo et al., 2020). In laboratory settings mice are typically separated from their mother by P21-P28, in our study at P21, which is considered the time of weaning. The transition to independent feeding marks a highly plastic time in the development of taste processing that can influence taste preferences later in life in humans (Mennella and Beauchamp, 2002; Trabulsi and Mennella, 2012) and mice (Schiff et al., 2023). A recent study in mice revealed that early experience with taste influences the maturation of inhibitory circuits mediated by PV+ INs in GC (Schiff et al., 2023). The results we present here indicate that during the period of transition to independent feeding, when mice experience tastes different from their mother's milk, there is extensive refinement of GC pyramidal neurons' excitability and PV+ INs circuits. This period of maturation extends into young adulthood".

Regarding the role of diet, it is possible that raising animals with special diets may induce changes in GC neuron properties. It is also possible that manipulating the time of weaning may affect the development of a variety of neural circuits (Terranova et al, 2001; Kanari et al, 2005; Kikusui and Mori, 2009; Richter et al, 2016; but see Bailoo et al, 2020). However, properly addressing these questions goes well beyond the scope of the present work.

The authors did not observe changes in intrinsic properties like AP threshold, rheobase or input resistance to be in line with the reduced I/F curve in P35 mice. However, the AP threshold (in Fig 1) does show a trend to be higher in P35 compared to P17 and P56. This would be in line with the reduced input/output function shown in Fig 1C. To better interpret the AP threshold, it would be important to also show the resting membrane potential and the difference between the two measurements. Was there any difference in resting membrane potential between these age ranges? In our experimental conditions, Vm does not directly affect the I/F curve because we hold all recorded neurons at -70mV upon establishing the current clamp mode (now included in the methods section lines 135-139). We do this to reduce the impact of variable resting membrane potentials on our measurements. In these conditions, the dynamic input resistance (Methods: "calculated as the slope of the current-voltage curve for steps below action potential threshold"), rheobase and action potential threshold are the primary parameters revealing changes in membrane excitability. We do measure the amount of current injected to hold neurons at -70mV (which was not significantly different between groups), but we did not include this parameter in the manuscript as the other parameters provide a more thorough description of membrane properties.

Minor comments Why did the authors focus on pyramidal neurons in deep infragranular layers, and how did they record from infragranular layers in all three divisions of GC (granular, dysgranular and agranular)? We edited the text to clarify this (lines: 286-298). "We focused on this population of neurons as they are primary output neurons of cortical circuits. Recordings were obtained primarily from the granular and dysgranular division of GC, with a few neurons recorded in agranular GC as well. The 3 divisions of GC can be identified under DIC optics within the same coronal slice by a tapering of layer 4. Hoechst counterstain was used to identify landmarks for borders between cortical regions and divisions. During post hoc image analysis, we applied a mask to each coronal section delineating the boundaries of GC and its subdivisions. This mask was created from sample sections that were immunostained for vGluT2, a marker of thalamic glutamatergic inputs which enabled visualization of layer 4, and Hoechst. Borders were manually traced around GC by comparing our stains and two mouse brain atlases (Paxinos &Franklin, 2001 and the Allen Mouse Brain Atlas). For images of each experimental slice, the mask was aligned laterally to the pial surface, medially to the corpus callosum, and ventrally to the rhinal fissure, allowing consistency across slices in determining into which subdivision the recorded cell belonged. As within each age group the parameters we measured showed no differences across divisions, data were pooled." Which electrophysiology recording system was used? Together with which software? Was series resistance compensation done by hand or automatically? We edited the Methods to include this information (lines 124-129). "Whole-cell patch clamp recordings were obtained from visually identified layer 5 pyramidal neurons under DIC optics using borosilicate glass pipettes with resistance of 3-5MΩ. The recording equipment included a HEKA amplifier (EPC10 Quadro USB; Heka Elektronik) with integrated data acquisition. Signals were sampled at 10KHz. Cell capacitance was not compensated during the recording. Patchmaster software (Heka Elektronik) was used for data acquisition".

Mice of both genders were used, but was there any sex difference in the results? No sex differences were observed in the parameters we measured within each age group, therefore data from male and female mice were pooled.

Line 83: What is the sensitive window for the development of sweet taste preference.

Schiff et al, 2023 Science Advances identified a sensitive window for the development of taste preference. The developmental window examined in the present study is based on that study.

Line 277, What was compared in two-way RM ANOVA (with main effect of age). Was gender also included as factor? It might be better to use linear mixed effect models for statistical testing to exclude effects of individual mice or gender As we did not observe any sex differences for any parameter we tested within each age group, we did not include sex as a factor in the RM ANOVA.

Fig 2: DAB staining is difficult to see These are images of thick (350µm) slices taken at 4x with a light microscope. At this magnification with such thick slices, it is not possible to define processes.

Higher magnification images of these same neurons were initially taken using confocal fluorescence microscopy and tiling before unmounting and reprocessing slices for DAB and are included in Fig. 1 and Fig. 3.

While the images in Fig. 2A cannot clearly show the detailed morphology of the neurons, they show that we were able to convert fluorescence staining to DAB. The reconstructions with the Neurolucida system were done by imaging neurons at 40x while the system adjusts the focus of the image with a motorized system to track labelled processes.

Fig 3a shows the same as Fig1a, just with a different subset. It might be better to show all recorded neurons in a supplementary figure.

The experiments in Fig. 1A and Fig. 3A were done using different internal solutions. Neurons shown in Fig. 1A were recorded using a potassium gluconate internal solution, therefore their identity was confirmed by their firing pattern and by immunohistochemistry. Neurons in Fig. 3A were recorded with a Cs2SO4 solution which included the Na channel blocker QX314. Firing patterns cannot be assessed with this internal solution, therefore the only way to confirm that the neurons included in the analysis of synaptic currents are indeed layer 5 pyramidal neurons in GC was the use of immunohistochemistry. Given the differences in experimental conditions, we feel it is important to show corresponding images in both figures.

Were recordings done in consecutive order (first -50mV then +10 mV)? Did order have an effect on recoding result or quality? We added experimental details to the Methods to address this concern (lines 154-165): "Recordings were obtained in voltage clamp with an internal solution containing in mM: 20 KCl, 100 Cs2SO4, 10 K-HEPES, 4 Mg-ATP, 0.3 Na-GTP, 10 Na-phosphocreatine, 0.2% biocytin (Vrev Cl- = -49.3mV). The pH was adjusted to 7.35 with KOH and the osmolarity was adjusted to 295mOsm with sucrose. The sodium channel blocker QX314 (3mM, Tocris Bioscience) was added to the internal solution to stabilize recordings during prolonged depolarizations. Spontaneous postsynaptic excitatory and inhibitory currents (sEPSCs and sIPSCs respectively) were recorded by holding neurons at three different holding potentials around the expected reversal for chloride (in mV: -55, -50, -45) and for AMPA/NMDA receptor mediated currents (in mV: +5, +10, +15). Post hoc current vs. holding voltage (Vhold) functions were used to identify the holding voltage that best isolated sEPSCs and sIPSCs respectively. Traces recorded at the optimal holding voltage were then used for analysis of amplitude and frequency of spontaneous events (Maffei et al., 2004; Maffei and Turrigiano, 2008b). The analysis of spontaneous current was done using Clampfit software (Molecular Devices)".

We have used this approach in several studies from different cortical regions (V1: Maffei et al, 2004, 2010; Kloc 2014; Tatti and Maffei, 2017; GC: Haley et al, 2016, 2020, 2023; Schiff et al, 2023; M1: Swanson et al, 2023). To date, we have not observed any effect of the order of the depolarization on quality of the recordings or results.

Line 341: better explain what is shown here. What are the medians of the frequency or amplitude? We apologize for not including average and standard error for the data. The manuscript has been edited to include these values.

Figure 5: Only 6 cells out of 3 mice recorded at p21. To be compared with p56. Unclear why p35 has not been used? The number of PV neurons in GC at P21 is much lower than in adult mice and the amplitude of evoked IPSCs is also much lower. We have included the number of recorded maps that reliably showed evoked responses so that we could compare these results to those obtained at P56.

As P35 is comparable to P17-P21 for some parameters and to P56 for others, we chose to focus on the earliest and latest age group to examine weanling and young adult PV+ INs connectivity.

Reviewer #2 This is a well-done inquiry into developmental changes in gustatory cortical circuitry, showing increases in the strength of PV inhibition onto pyramidal cells between P17 and P56. The authors' most central effect (Figure 3H) is very cool, and itself well worthy of publication. I also like the scholarliness of the comparisons to visual cortical development. While I have many suggestions on how to improve the manuscript, all will be easily handled by the authors, and none dampen my enthusiasm for the work. Those suggestions follow.

The effects are intriguingly but annoyingly varied. In one case, a P17-P35 difference resets by P56, but in other cases it doesn't, and in still other cases change only occurs after P35. Again, this is fascinating, and it also really drives home the authors' point about something happening in the general vicinity of P35 (more on that below), but it is NOT clear that the effects being observed are related to one another. The authors should either provide a conceptual framework whereby the diverse timings of effects can be thought of as part of a single developmental process (e.g., explaining why an earlier change might be thought of as leading to a later change) or be a little more circumspect in their prose (e.g., explicitly discussing the possibility that independent changes are happening at different times).

Thank you for the positive comments and for raising an important point. The current data set does not allow us to show a causal relationship among the changes we observed. We relied on scientific literature from other sensory systems, where many more studies of postnatal developmental circuit changes have been produced by our group and others, to interpret our data and provide hypotheses for how the changes we observed may fit together. Future work is needed to test whether the developmental changes in membrane properties, intrinsic and synaptic, and circuit connectivity depend on one another. We have edited the text to be more explicit about our goals and the limitations of our interpretations.

Given that mouse pup weaning happens in the 3rd week of life, I'm a bit confused as to why the authors didn't test closer to that date. While the authors' data definitely seem to suggest nonlinearities of change, in that things are stable across 2 out of 3 time points and "suddenly" change across one time point and the 3rd, it is not at all clear that these changes are tightly linked to weaning. This particularly true of the changes that occur after P35. I suggest that the authors lean away from organizing their Intro around weaning, a change that would have the added benefit of making the analogy with visual development more compelling.

We apologize if we gave the impression that weaning may be a tightly timed process. We have edited the introduction to include background information about the development of the gustatory system that guided our choice of age groups and clarify our goals (lines 36-49). "During postnatal development, mammals shift from relying on their mother's milk to foraging for food. Early experience with feeding independence influences the development of taste preferences (Schiff et al., 2023). While the postnatal development of gustatory cortical circuits is not well studied, there is some experimental evidence for protracted maturation of neuronal morphology and early-life experience-dependent effects on neurons in other regions of the taste system. In mice, taste receptor cells begin to reliably fire action potentials during the third postnatal week (Bigiani et al., 2002) and the refinement of their excitability extends into adulthood (Bigiani et al., 2002; Ohtubo et al., 2012). Postnatal anatomical re-wiring was observed in the first central relay in the gustatory system, the nucleus of the solitary tract (NTS) after postnatal day 21 (P21), with the inputs to the NTS reaching adult connectivity by P35 and undergoing additional refinement into adulthood (Hill et al., 1983; Sollars et al., 2006; May et al., 2008; Sun et al., 2017). In the gustatory portion of the parabrachial nucleus, dendritic arborization of multipolar and fusiform cells reach adult morphology by P35 (Lasiter and Kachele, 1988). These studies identify the postnatal window between P15-P21, P21-P35, and P50-P65 as periods of maturation for different circuits in the gustatory system".

We have also edited the discussion to include information about the weaning process in mice and the corresponding references (lines 657-669). "Mice begin their transition toward eating solid food around P12-P14 and by P17-P21 they become fully independent eaters (Bechard and Mason, 2010; Bailoo et al., 2020). In laboratory settings mice are typically separated from their mother by P21-P28, in our study at P21, which is considered the time of weaning. The transition to independent feeding marks a highly plastic time in the development of taste processing that can influence taste preferences later in life in humans (Mennella and Beauchamp, 2002; Trabulsi and Mennella, 2012) and mice (Schiff et al., 2023). A recent study in mice revealed that early experience with taste influences the maturation of inhibitory circuits mediated by PV+ INs in GC (Schiff et al., 2023). The results we present here indicate that during the period of transition to independent feeding, when mice experience tastes different from their mother's milk, there is extensive refinement of GC pyramidal neurons' excitability and PV+ INs circuits. This period of maturation extends into young adulthood".

Relatedly, I see little evidence for gradual changes in the authors' data. Given their sampling, it looks as if for virtually every effect, the data are stable across 2 of 3 time points and different for the 3rd. The authors should either remove language like "progressive decrease" (Line 288), which suggests similar amounts of change between P17 and P35 and between P35 and P56, or else do a better job of motivating that language (by, e.g., performing an analysis that shows that change to be truly gradual).

We understand the reviewer's concern and have edited the text as suggested.

In Lines 321-325, the authors suggest that the lengthening of the apical dendrite cannot explain the effects described in Figures 1 &2. I believe them, but it would be a good idea to provide some sort of references to work (theoretical work, even) that makes it clear what physiological changes do and don't follow from lengthening of the apical dendrite.

We have edited the discussion to address this issue (lines 614-623) "The increase in apical dendrite length together with the augmented cell surface are expected to increase membrane capacitance, an effect that could explain the decrease in input resistance observed at P56 (Marty and Neher, 1995). In addition, a longer dendrite may explain the decreased sEPSC amplitude (Segev and London, 2000). However, increased cell capacitance and decreased input resistance are not consistent with the observation that the P56 input/output function is restored to P17 levels in the absence of changes in rheobase and action potential threshold across age groups. Furthermore, the apical dendrite elongation does not explain the increase in sIPSCs amplitude, nor the decrease in sIPSC decay time constant. An increase in cell surface and longer dendritic processes predict the opposite effects in the absence of other factors such as changes in connectivity, recruitment of active conductance or plasticity (Segev and London, 2000; Chklovskii et al., 2004)." I missed a technical description of how the authors "sorted PV+ 407 INs into 2 groups, one without an associated PNN and one with a PNN." Particularly given my suspicion that some PNNs are less clear than others, the authors should provide a thorough explanation of this analysis (my apologies if they did so and I just missed it).

We apologize for not including these important details. The text in the Methods and in the Results has been revised accordingly.

Methods (Lines 262-267). "The group of PV+ neurons with a PNN included both neurons' somata surrounded by mature, high WFA signal intensity PNNs, and immature, low WFA signal intensity PNNs. The group of PV+ INs lacking PNNs did not show any above-background WFA signal around the somata. The intensity of PV and WFA fluorescence signals were quantified for each ROI in each preparation, normalized to the respective slice background fluorescence and plotted to test for possible correlation between the two parameters in each age group.".

Results: (Lines 459-469). "Neurons were defined as PV+ IN if they showed co-localization of the Neurotrace signal with the PV signal. They were also identified as associated with a PNN if the PV and Neurotrace signals were surrounded by clustered WFA fluorescence. The PV+ IN with PNN included PV neurons with mature (high intensity and accumulation) and immature (low intensity and more diffuse cluster of fluorescence) around the somata (Fig. 4A). PV+ IN without a PNN were defined as neurons showing co-localization of the Neurotrace and PV signals whose soma was not surrounded by WFA fluorescence. The fluorescence signal was quantified as distribution of intensity vs cell number for each age group (Fig. 4C-E). For statistical comparisons across slices and animals, the background fluorescence intensity (measured as the average intensity of the entire GC for each imaging channel) was subtracted from the corresponding channel fluorescence intensity for each identified PV and WFA ROI.".

I have some concerns about the analysis summarized in Figure 4F. I assume that the histology varied in quality (i.e., in general brightness) from mouse to mouse and even slice to slice, and this general variability could cause an artifactual correlation between the brightness of two imaged variables. It would be valuable to demonstrate that this confound did not impact results, either by: 1) adding in a 3rd, unrelated variable and showing that it was uncorrelated with the other two; or 2) doing a within-slice analysis showing that even with any between variability nullified the effect remains.

Thank you for raising this important point. We have edited the Methods and the Results to address this issue.

Methods (Lines 250-259): "Quantification of the number of PV+ INs and PNNs as well as of fluorescence intensity was obtained in ImageJ using the Pipsqueak AI macro from Rewire Neuro, Inc (Sorg et al., 2016). Individual ROIs for each PV+ neuron or PNN were automatically created and verified by an experimenter who was blind to the experimental condition. Images from the different fluorescence channels were analyzed separately and then the X and Y coordinates of each ROI were used to determine overlap, which was then confirmed by visual inspection. All single- or double-labeled neurons or PNNs were detected and the mean fluorescence intensity within the ROI was measured. For each image, the average fluorescence intensity across the entire GC region was calculated and was used as background value. The background fluorescence value was subtracted from each ROI fluorescence intensity signal before statistical analysis. This procedure was used to normalize measurements across sections and preparations".

Methods (Lines 490-492): "To do that, PV fluorescence intensity normalized to the corresponding slice Neurotrace signal for each cell was plotted against each cell's normalized WFA signal (Fig. 4F) and their correlation quantified with a Spearman correlation test".

It is not clear to me how the Figure 5D-F results, which show reductions of connectivity with age, are properly interpreted to suggest that "PV+ INs input onto GC pyramidal neurons becomes more spatially restricted over the course 476 of postnatal development" (Lines 474-477). The authors need to either more clearly explain or remove that claim.

We rewrote the entire section to clarify the results and their interpretation (Lines 522-562). We do not report the text changes here because they are too extensive.

The writing could still use some work. A non-exhaustive list of the issues that I observed includes:

1) Repeated redundancies involving the word "dynamic." As the definition of the word "dynamic" is "changing," expressions like "dynamic changes" means "changing changes." Even expressions such as "dynamic circuit refinement" (Lines 81-82) are subtly redundant.

Thank you for pointing out these issues. The text has been edited to address them to the best of our abilities.

2) The authors should choose to refer either to "sensitive" and "critical" periods, and not switch between those terms (unless they explicitly explain why they are doing so).

The text was edited for consistency.

3) Lengthy descriptions of non-significant effects (e.g., Lines 363-366) should be omitted.

Corrected.

4) The authors start a lot of sentences with a single word, followed by a comma, and followed by the rest of the sentence. While commonly used, this is an often awkward and often inappropriate construction, and its use should be minimized. Most notably, the authors should remove the two paragraphs in a row that start with "Finally,..." (Lines 428 and 436).

Edited to correct this.

5) There are a number of agrammaticisms.

We did our best to address them all.

Finally, I have quite a few small Figure issues:

- In general, panels in some figures read reliably increasing in rows from left to right, whereas in other figures, panel ordering suddenly switches to being columnar, from top to bottom. This is very confusing, and should be changed.

We did our best to rearrange figure panels while maintaining readability of the figures and consistency with the text.

- For Figure 1D, I'd like x-axis labels despite the colors (which refer to a different panel). Also, I recommend removing 1E-G, which show nothing, so that you can make 1D larger.

We did our best to address this - Figure 2D &E could be combined, again allowing the authors to avoid presenting entire panels that show no effects.

We understand the concern of the reviewer but in this case negative results help with the data interpretation, and we feel they should be included in the figure.

- Figure 3A seems to show what was already shown in Figure 1A. It should be removed, because the reader doesn't need to be convinced again that pyramidal cells can be identified that aren't interneurons. Furthermore, the effect in Figure 3D ("opposite age-dependent changes in frequency and amplitude") is non-compelling as presented.

The experiments in Fig. 1A and Fig. 3A were done using different internal solutions. Neurons shown in Fig. 1A were recorded using a potassium gluconate internal solution, therefore their identity was confirmed by their firing pattern and by immunohistochemistry. Neurons in Fig. 3A were recorded with a Cs2SO4 solution which included the Na channel blocker QX314. Firing patterns cannot be assessed with this internal solution, therefore the only way to confirm that the neurons included in the analysis of synaptic currents are indeed layer 5 pyramidal neurons in GC was the use of immunohistochemistry. Given the differences in experimental conditions, we feel it is important to show corresponding images in both figures.

- Figure 4C is also unclear. I assume that the effect to which the authors are referring in Lines 410-411 has to do with how far to the right the cumulative sum plots go for the different samples. If so, the authors need to very explicitly point out that this is the nature of the effect. A better idea, however, would be to actually present the data as distributions (i.e., frequency rather than cumulative frequency in the y-axis).

As suggested by the reviewer, the cumulative distributions have been substituted with histograms to better visualize the results.

## References

[B1] Antonini A, Fagiolini M, Stryker MP (1999) Anatomical correlates of functional plasticity in mouse visual cortex. J Neurosci 19:4388–4406. 10.1523/JNEUROSCI.19-11-04388.1999 10341241 PMC2452998

[B2] Baho E, et al. (2019) P75 neurotrophin receptor activation regulates the timing of the maturation of cortical parvalbumin interneuron connectivity and promotes juvenile-like plasticity in adult visual cortex. J Neurosci 39:4489–4510. 10.1523/JNEUROSCI.2881-18.2019 30936240 PMC6554620

[B3] Bailoo JD, Voelkl B, Varholick J, Novak J, Murphy E, Rosso M, Palme R, Wurbel H (2020) Effects of weaning age and housing conditions on phenotypic differences in mice. Sci Rep 10:11684. 10.1038/s41598-020-68549-3 32669633 PMC7363894

[B4] Bechard A, Mason G (2010) Leaving home: a study of laboratory mouse pup independence. Appl Anim Behav Sci 125:181–188. 10.1016/j.applanim.2010.04.006

[B5] Berardi N, Pizzorusso T, Ratto GM, Maffei L (2003) Molecular basis of plasticity in the visual cortex. Trends Neurosci 26:369–378. 10.1016/S0166-2236(03)00168-1 12850433

[B6] Bigiani A, Cristiani R, Fieni F, Ghiaroni V, Bagnoli P, Pietra P (2002) Postnatal development of membrane excitability in taste cells of the mouse vallate papilla. J Neurosci 22:493–504. 10.1523/JNEUROSCI.22-02-00493.2002 11784795 PMC6758677

[B7] Chattopadhyaya B, Di Cristo G, Higashiyama H, Knott GW, Kuhlman SJ, Welker E, Huang ZJ (2004) Experience and activity-dependent maturation of perisomatic GABAergic innervation in primary visual cortex during a postnatal critical period. J Neurosci 24:9598–9611. 10.1523/JNEUROSCI.1851-04.2004 15509747 PMC6730138

[B8] Chattopadhyaya B, Di Cristo G, Wu CZ, Knott G, Kuhlman S, Fu Y, Palmiter RD, Huang ZJ (2007) GAD67-mediated GABA synthesis and signaling regulate inhibitory synaptic innervation in the visual cortex. Neuron 54:889–903. 10.1016/j.neuron.2007.05.015 17582330 PMC2077924

[B9] Chklovskii DB, Mel BW, Svoboda K (2004) Cortical rewiring and information storage. Nature 431:782–788. 10.1038/nature03012 15483599

[B10] Ciganok-Huckels N, Jehasse K, Kricsfalussy-Hrabar L, Ritter M, Ruland T, Kampa BM (2023) Postnatal development of electrophysiological and morphological properties in layer 2/3 and layer 5 pyramidal neurons in the mouse primary visual cortex. Cereb Cortex 33:5875–5884. 10.1093/cercor/bhac467 36453454 PMC10183751

[B11] Clements JD, Bekkers JM (1997) Detection of spontaneous synaptic events with an optimally scaled template. Biophys J 73:220–229. 10.1016/S0006-3495(97)78062-7 9199786 PMC1180923

[B12] Desai NS, Cudmore RH, Nelson SB, Turrigiano GG (2002) Critical periods for experience-dependent synaptic scaling in visual cortex. Nat Neurosci 5:783–789. 10.1038/nn878 12080341

[B13] Donato F, Rompani SB, Caroni P (2013) Parvalbumin-expressing basket-cell network plasticity induced by experience regulates adult learning. Nature 504:272–276. 10.1038/nature12866 24336286

[B14] Elston GN, Fujita I (2014) Pyramidal cell development: postnatal spinogenesis, dendritic growth, axon growth, and electrophysiology. Front Neuroanat 8:78. 10.3389/fnana.2014.00078 25161611 PMC4130200

[B15] Fagiolini M, Hensch TK (2000) Inhibitory threshold for critical-period activation in primary visual cortex. Nature 404:183–186. 10.1038/35004582 10724170

[B17] Gainey MA, Feldman DE (2017) Multiple shared mechanisms for homeostatic plasticity in rodent somatosensory and visual cortex. Philos Trans R Soc Lond B Biol Sci 372:20160157. 10.1098/rstb.2016.0157 28093551 PMC5247589

[B16] Gainey MA, Aman JW, Feldman DE (2018) Rapid disinhibition by adjustment of PV intrinsic excitability during whisker map plasticity in mouse S1. J Neurosci 38:4749–4761. 10.1523/JNEUROSCI.3628-17.2018 29678876 PMC5956988

[B18] Gogolla N, Takesian AE, Feng G, Fagiolini M, Hensch TK (2014) Sensory integration in mouse insular cortex reflects GABA circuit maturation. Neuron 83:894–905. 10.1016/j.neuron.2014.06.033 25088363 PMC4177076

[B19] Gonchar Y, Wang Q, Burkhalter A (2007) Multiple distinct subtypes of GABAergic neurons in mouse visual cortex identified by triple immunostaining. Front Neuroanat 1:3. 10.3389/neuro.05.003.2007 18958197 PMC2525923

[B20] Haley MS, Fontanini A, Maffei A (2016) Laminar- and target-specific amygdalar inputs in rat primary gustatory cortex. J Neurosci 36:2623–2637. 10.1523/JNEUROSCI.3224-15.2016 26937004 PMC4879210

[B21] Haley MS, Fontanini A, Maffei A (2023) Inhibitory gating of thalamocortical inputs onto rat gustatory insular cortex. J Neurosci 43:7294–7306. 10.1523/JNEUROSCI.2255-22.2023 37704374 PMC10621769

[B22] Heinen K, Bosman LW, Spijker S, van Pelt J, Smit AB, Voorn P, Baker RE, Brussaard AB (2004) GABAA receptor maturation in relation to eye opening in the rat visual cortex. Neuroscience 124:161–171. 10.1016/j.neuroscience.2003.11.004 14960348

[B23] Hensch TK (2004) Critical period regulation. Annu Rev Neurosci 27:549–579. 10.1146/annurev.neuro.27.070203.144327 15217343

[B24] Hill DL, Bradley RM, Mistretta CM (1983) Development of taste responses in rat nucleus of solitary tract. J Neurophysiol 50:879–895. 10.1152/jn.1983.50.4.879 6631468

[B25] Kroon T, van Hugte E, van Linge L, Mansvelder HD, Meredith RM (2019) Early postnatal development of pyramidal neurons across layers of the mouse medial prefrontal cortex. Sci Rep 9:5037. 10.1038/s41598-019-41661-9 30911152 PMC6433913

[B26] Kubota Y, Karube F, Nomura M, Kawaguchi Y (2016) The diversity of cortical inhibitory synapses. Front Neural Circuits 10:27. 10.3389/fncir.2016.00027 27199670 PMC4842771

[B27] Lasiter PS, Kachele DL (1988) Postnatal development of the parabrachial gustatory zone in rat: dendritic morphology and mitochondrial enzyme activity. Brain Res Bull 21:79–94. 10.1016/0361-9230(88)90122-0 2464423

[B28] Lo SQ, Sng JCG, Augustine GJ (2017) Defining a critical period for inhibitory circuits within the somatosensory cortex. Sci Rep 7:7271. 10.1038/s41598-017-07400-8 28779074 PMC5544762

[B29] Lupori L, et al. (2023) A comprehensive atlas of perineuronal net distribution and colocalization with parvalbumin in the adult mouse brain. Cell Rep 42:112788. 10.1016/j.celrep.2023.112788 37436896

[B30] Madisen L, et al. (2010) A robust and high-throughput Cre reporting and characterization system for the whole mouse brain. Nat Neurosci 13:133–140. 10.1038/nn.2467 20023653 PMC2840225

[B34] Maffei A, Turrigiano GG (2008a) Multiple modes of network homeostasis in visual cortical layer 2/3. J Neurosci 28:4377–4384. 10.1523/JNEUROSCI.5298-07.2008 18434516 PMC2655203

[B35] Maffei A, Turrigiano GG (2008b) The age of plasticity: developmental regulation of synaptic plasticity in neocortical microcircuits. Prog Brain Res 169:211–223. 10.1016/S0079-6123(07)00012-X 18394476

[B31] Maffei A, Nelson SB, Turrigiano GG (2004) Selective reconfiguration of layer 4 visual cortical circuitry by visual deprivation. Nat Neurosci 7:1353–1359. 10.1038/nn1351 15543139

[B32] Maffei A, Nataraj K, Nelson SB, Turrigiano GG (2006) Potentiation of cortical inhibition by visual deprivation. Nature 443:81–84. 10.1038/nature05079 16929304

[B33] Maffei A, Lambo ME, Turrigiano GG (2010) Critical period for inhibitory plasticity in rodent binocular V1. J Neurosci 30:3304–3309. 10.1523/JNEUROSCI.5340-09.2010 20203190 PMC2848504

[B36] Marty A, Neher E (1995) Tight-seal whole-cell recordings. In: *Single channel recording*, 2nd ed. (Sakmann B, Neher E, eds), pp 31–52. New York: Plenum Press/Springer.

[B37] May OL, Erisir A, Hill DL (2008) Modifications of gustatory nerve synapses onto nucleus of the solitary tract neurons induced by dietary sodium-restriction during development. J Comp Neurol 508:529–541. 10.1002/cne.21708 18366062 PMC2596877

[B38] Mennella JA, Beauchamp GK (2002) Flavor experiences during formula feeding are related to preferences during childhood. Early Hum Dev 68:71–82. 10.1016/S0378-3782(02)00008-7 12113993 PMC2987582

[B39] Micheva KD, Beaulieu C (1995) Postnatal development of GABA neurons in the rat somatosensory barrel cortex: a quantitative study. Eur J Neurosci 7:419–430. 10.1111/j.1460-9568.1995.tb00338.x 7773439

[B40] Murase S, Lantz CL, Quinlan EM (2017) Light reintroduction after dark exposure reactivates plasticity in adults via perisynaptic activation of MMP-9. Elife 6:e27345. 10.7554/eLife.27345 28875930 PMC5630258

[B41] Ohtubo Y, Iwamoto M, Yoshii K (2012) Subtype-dependent postnatal development of taste receptor cells in mouse fungiform taste buds. Eur J Neurosci 35:1661–1671. 10.1111/j.1460-9568.2012.08068.x 22462540

[B42] Paxinos G, Franklin KBJ (2001) *The mouse brain in stereotaxic coordinates*, Ed 2. San Diego: Academic Press.

[B43] Petreanu L, Huber D, Sobczyk A, Svoboda K (2007) Channelrhodopsin-2-assisted circuit mapping of long-range callosal projections. Nat Neurosci 10:663–668. 10.1038/nn1891 17435752

[B44] Pizzorusso T, Medini P, Berardi N, Chierzi S, Fawcett JW, Maffei L (2002) Reactivation of ocular dominance plasticity in the adult visual cortex. Science 298:1248–1251. 10.1126/science.1072699 12424383

[B45] Santos-Silva T, Colodete DAE, Lisboa JRF, Silva Freitas I, Lopes CFB, Hadera V, Lima TSA, Souza AJ, Gomes FV (2024) Perineuronal nets as regulators of parvalbumin interneuron function: factors implicated in their formation and degradation. Basic Clin Pharmacol Toxicol 134:614–628. 10.1111/bcpt.13994 38426366

[B46] Schiff HC, Kogan JF, Isaac M, Czarnecki LA, Fontanini A, Maffei A (2023) Experience-dependent plasticity of gustatory insular cortex circuits and taste preferences. Sci Adv 9:eade6561. 10.1126/sciadv.ade6561 36630501 PMC9833665

[B47] Segev I, London M (2000) Untangling dendrites with quantitative models. Science 290:744–750. 10.1126/science.290.5492.744 11052930

[B48] Shemesh OA, Tanese D, Zampini V, Linghu C, Piatkevich K, Ronzitti E, Papagiakoumou E, Boyden ES, Emiliani V (2017) Temporally precise single-cell-resolution optogenetics. Nat Neurosci 20:1796–1806. 10.1038/s41593-017-0018-8 29184208 PMC5726564

[B49] Slaker ML, Harkness JH, Sorg BA (2016) A standardized and automated method of perineuronal net analysis using Wisteria floribunda agglutinin staining intensity. IBRO Rep 1:54–60. 10.1016/j.ibror.2016.10.001 28713865 PMC5507617

[B50] Sollars SI, Walker BR, Thaw AK, Hill DL (2006) Age-related decrease of the chorda tympani nerve terminal field in the nucleus of the solitary tract is prevented by dietary sodium restriction during development. Neuroscience 137:1229–1236. 10.1016/j.neuroscience.2005.09.040 16338076 PMC4965233

[B51] Sorg BA, Berretta S, Blacktop JM, Fawcett JW, Kitagawa H, Kwok JC, Miquel M (2016) Casting a wide net: role of perineuronal nets in neural plasticity. J Neurosci 36:11459–11468. 10.1523/JNEUROSCI.2351-16.2016 27911749 PMC5125213

[B52] Southwell DG, Froemke RC, Alvarez-Buylla A, Stryker MP, Gandhi SP (2010) Cortical plasticity induced by inhibitory neuron transplantation. Science 327:1145–1148. 10.1126/science.1183962 20185728 PMC3164148

[B53] Sun C, Hummler E, Hill DL (2017) Selective deletion of sodium salt taste during development leads to expanded terminal fields of gustatory nerves in the adult mouse nucleus of the solitary tract. J Neurosci 37:660–672. 10.1523/JNEUROSCI.2913-16.2016 28100747 PMC5242412

[B54] Takesian AE, Kotak VC, Sanes DH (2012) Age-dependent effect of hearing loss on cortical inhibitory synapse function. J Neurophysiol 107:937–947. 10.1152/jn.00515.2011 22090457 PMC3289466

[B55] Takesian AE, Bogart LJ, Lichtman JW, Hensch TK (2018) Inhibitory circuit gating of auditory critical-period plasticity. Nat Neurosci 21:218–227. 10.1038/s41593-017-0064-2 29358666 PMC5978727

[B56] Taniguchi H, et al. (2011) A resource of Cre driver lines for genetic targeting of GABAergic neurons in cerebral cortex. Neuron 71:995–1013. 10.1016/j.neuron.2011.07.026 21943598 PMC3779648

[B57] Tatti R, Swanson OK, Lee MSE, Maffei A (2017) Layer-specific developmental changes in excitation and inhibition in rat primary visual cortex. eNeuro 4:ENEURO.0402-17.2017. 10.1523/ENEURO.0402-17.2017 PMC5779119 29379869

[B58] Trabulsi JC, Mennella JA (2012) Diet, sensitive periods in flavour learning, and growth. Int Rev Psychiatry 24:219–230. 10.3109/09540261.2012.675573 22724643 PMC4575790

[B59] Wang L, Fontanini A, Maffei A (2011) Visual experience modulates spatio-temporal dynamics of circuit activation. Front Cell Neurosci 5:12. 10.3389/fncel.2011.00012 21743804 PMC3127086

[B60] Wu X, Fu Y, Knott G, Lu J, Di Cristo G, Huang ZJ (2012) GABA signaling promotes synapse elimination and axon pruning in developing cortical inhibitory interneurons. J Neurosci 32:331–343. 10.1523/JNEUROSCI.3189-11.2012 22219294 PMC3742883

[B61] Yamamoto T (1984) Taste responses of cortical neurons. Prog Neurobiol 23:273–315. 10.1016/0301-0082(84)90007-8 6398454

[B62] Zucker RS, Regehr WG (2002) Short-term synaptic plasticity. Annu Rev Physiol 64:355–405. 10.1146/annurev.physiol.64.092501.114547 11826273

