# Supplementary figures and images for: Postnatal Development of Pyramidal Neurons Excitability and Synaptic Inputs in Mouse Gustatory Cortical Circuits
Source: eNeuro. 2026 Apr 28;13(4):ENEURO.0329-25.2026. doi: 10.1523/ENEURO.0329-25.2026 (PMC13124029; doi:10.1523/ENEURO.0329-25.2026)

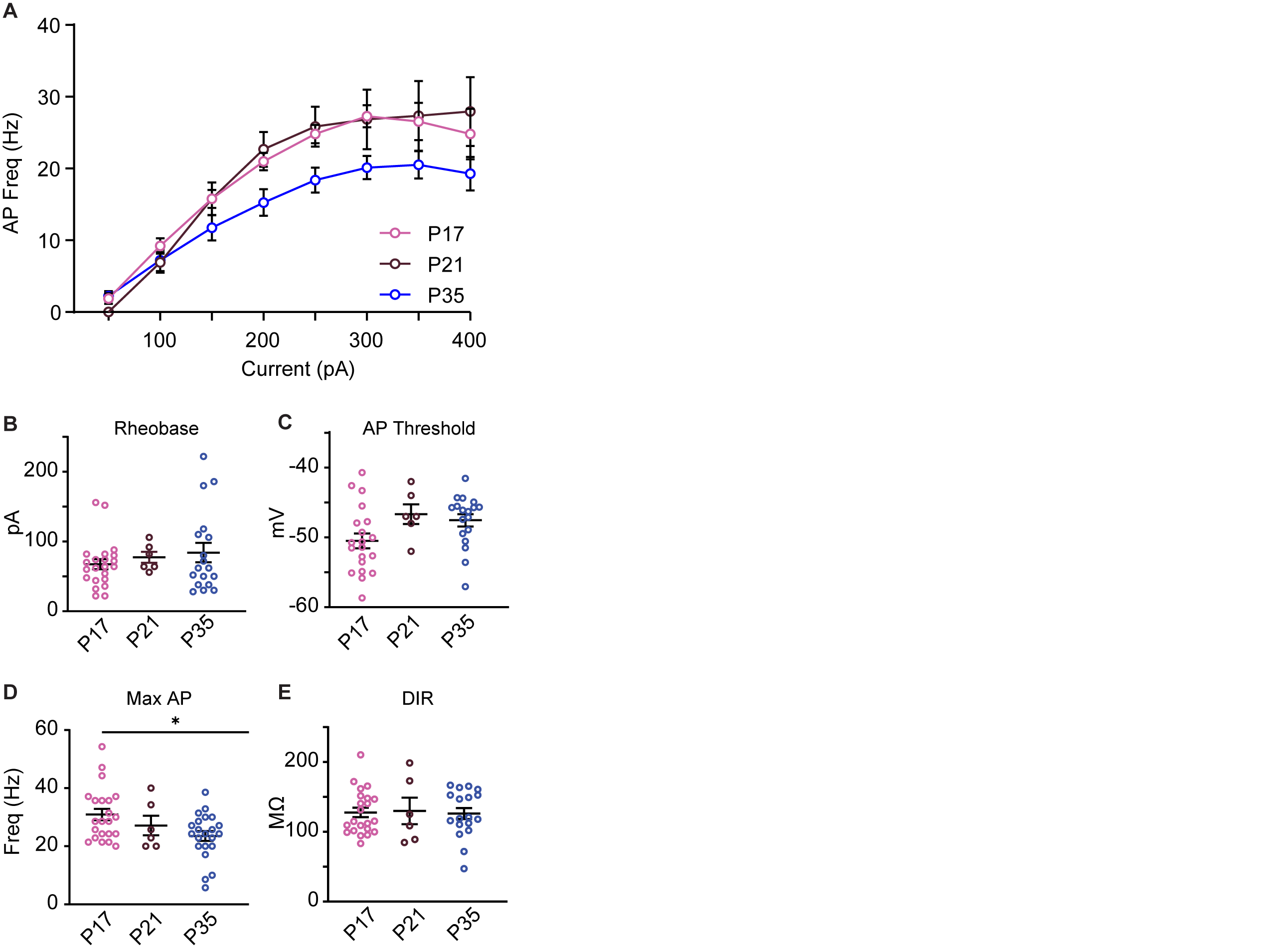

Supplement: Figure 5-1 — Postnatal developmental changes in intrinsic excitability of GC layer 5 pyramidal neurons. A. Average input/output function for neurons recorded at P17 (pink), P21 (black) and P35 (blue). Data presented as mean ± SEM. B. Left. Rheobase for each recorded neuron in each age group. Right. Action potential (AP) threshold for each recorded neuron by age group. C. Left. Maximum firing frequency for all recorded neurons plotted by age group. Right. Dynamic input resistance (DIR) quantified as the slope of the input/output function for steps below rheobase. Data are reported as average ± SEM. Pink: P17; black: P21; blue: P35. The data for P17 and P35 are the same as in Fig. 1. Asterisks: p ≤ 0.05. Download Figure 5-1, TIF file. [file eneuro-13-ENEURO.0329-25.2026-s002.tif]
